# Impact of COVID-19 pandemic on depression incidence and healthcare service use among patients with depression: an interrupted time-series analysis from a 9-year population-based study

**DOI:** 10.1186/s12916-024-03386-z

**Published:** 2024-04-22

**Authors:** Vivien Kin Yi Chan, Yi Chai, Sandra Sau Man Chan, Hao Luo, Mark Jit, Martin Knapp, David Makram Bishai, Michael Yuxuan Ni, Ian Chi Kei Wong, Xue Li

**Affiliations:** 1 Department of Pharmacology and Pharmacy, Li Ka Shing Faculty of Medicine, The University of Hong Kong, Hong Kong SAR, China; 2 The Hong Kong Jockey Club Centre for Suicide Research and Prevention, The University of Hong Kong, Hong Kong SAR, China; 3 Department of Psychiatry, Faculty of Medicine, The Chinese University of Hong Kong, Hong Kong SAR, China; 4 Department of Social Work and Social Administration, Faculty of Social Sciences, The University of Hong Kong, Hong Kong SAR, China; 5 Department of Infectious Disease Epidemiology, London School of Hygiene & Tropical Medicine, London, UK; 6 Care Policy and Evaluation Centre, Department of Health Policy, London School of Economics and Political Science, London, UK; 7 School of Public Health, Li Ka Shing Faculty of Medicine, The University of Hong Kong, Hong Kong SAR, China; 8 The State Key Laboratory of Brain and Cognitive Sciences, The University of Hong Kong, Hong Kong SAR, China; 9 Urban Systems Institute, The University of Hong Kong, Hong Kong SAR, China; 10 Laboratory of Data Discovery for Health (D24H), Hong Kong SAR, China; 11 School of Pharmacy, Aston University, London, UK; 12 Department of Medicine, School of Clinical Medicine, Li Ka Shing Faculty of Medicine, The University of Hong Kong, Hong Kong SAR, China; 13 Advanced Data Analytics for Medical Science (ADAMS) Limited, Hong Kong SAR, China

**Keywords:** Depression, Incidence, Health resource utilization, Health service use, COVID, Pandemic, Interrupted time series, Electronic medical records, Cohort study

## Abstract

**Background:**

Most studies on the impact of the COVID-19 pandemic on depression burden focused on the earlier pandemic phase specific to lockdowns, but the longer-term impact of the pandemic is less well-studied. In this population-based cohort study, we examined the short-term and long-term impacts of COVID-19 on depression incidence and healthcare service use among patients with depression.

**Methods:**

Using the territory-wide electronic medical records in Hong Kong, we identified all patients aged ≥ 10 years with new diagnoses of depression from 2014 to 2022. We performed an interrupted time-series (ITS) analysis to examine changes in incidence of medically attended depression before and during the pandemic. We then divided all patients into nine cohorts based on year of depression incidence and studied their initial and ongoing service use patterns until the end of 2022. We applied generalized linear modeling to compare the rates of healthcare service use in the year of diagnosis between patients newly diagnosed before and during the pandemic. A separate ITS analysis explored the pandemic impact on the ongoing service use among prevalent patients with depression.

**Results:**

We found an immediate increase in depression incidence (RR = 1.21, 95% CI: 1.10–1.33, *p* < 0.001) in the population after the pandemic began with non-significant slope change, suggesting a sustained effect until the end of 2022. Subgroup analysis showed that the increases in incidence were significant among adults and the older population, but not adolescents. Depression patients newly diagnosed during the pandemic used 11% fewer resources than the pre-pandemic patients in the first diagnosis year. Pre-existing depression patients also had an immediate decrease of 16% in overall all-cause service use since the pandemic, with a positive slope change indicating a gradual rebound over a 3-year period.

**Conclusions:**

During the pandemic, service provision for depression was suboptimal in the face of increased demand generated by the increasing depression incidence during the COVID-19 pandemic. Our findings indicate the need to improve mental health resource planning preparedness for future public health crises.

**Supplementary Information:**

The online version contains supplementary material available at 10.1186/s12916-024-03386-z.

## Background

The COVID-19 pandemic that began in 2020 has resulted in an unprecedented public health crisis, with 771 million confirmed cases and over 6 million deaths across the globe as of September 2023 [1]. To curb the spread and reduce the mortality of SARS-CoV-2 infections, governments worldwide enacted stringent measures to contain its spread, including social mobility restrictions, mask-wearing, massive screenings, and lockdowns. Despite their effectiveness in limiting viral spread, these measures may have created a macro-environment of fear, social exclusion of individuals who contracted the virus, and reduced community cohesion [2–4]. The pandemic and the ensuing measures also led to economic disruption and created financial hardship for millions of families [4, 5]. The combined pandemic stresses may have exacerbated the risk factors for mental health conditions including depression. Among patients with pre-existing depression, the government effort re-prioritized for outbreak control may have also led to disrupted non-emergency services and unmet care need in mental health [6].

A meta-analysis estimated an additional 53 million cases of depression and a 27.6% increase in its global prevalence in 2020 due to COVID-19-related illnesses and reduced mobility [7], which affected individuals across age groups [8–10]. In Hong Kong, a survey showed a consistent mental health crisis with a two-fold increase in depression symptoms and a 28.3% rise in the stress level even during the well-managed small-scale outbreaks [11]. Conversely, other studies reported that the pandemic reduced the risk of depression and self-harm because of the emotional security provided by timely government intervention, but these findings were confounded by increased barriers to seek medical help [12–14]. In the emergency phase of the pandemic, it was reported that lockdowns significantly reduced healthcare service use for both outpatient and inpatient services [15–17]. Studies also found an elevated risk of depression relapse and use of antidepressants [18, 19].

Literature exploring pandemic impact on depression has mostly focused on the earlier phase of the pandemic (2020–2021) when short-term lockdown orders were in place. There are fewer studies and more mixed results for the post-emergency phase. Hong Kong followed the “dynamic zero-COVID policy” of China with strict border control, contact tracing, and quarantine before cases spread until the end of 2022 and so recorded a low number of SARS-CoV-2 cases for most of the time before a major Omicron outbreak [20]. It did not experience full lockdown, although stringent infection control and social measures were deployed for an entire 3-year-long period. This context thus enables us to evaluate the longer-term pandemic impact apart from a focus on lockdowns. In the late pandemic period, it is also useful to understand any potential decline in depression incidence and rebound in health service utilization. Using interrupted time series (ITS) analysis with a cohort study, we examined the changes in depression incidence and healthcare service use due to the pandemic, aiming to measure both the short-term (immediately after pandemic onset) and long-term (3 years since the outbreak) impacts on the burden of depression. We aimed to facilitate better preparedness in mental health resource planning for future public health crises.

## Methods

### Data source

We analyzed the Clinical Data Analysis and Reporting System (CDARS), the territory-wide routine electronic medical record (EMR) developed by the Hospital Authority, which manages all public healthcare services in Hong Kong and provides publicly funded healthcare services to all eligible residents (> 7.6 million). CDARS covers real-time anonymized patient-level data, including demographics, deaths, attendances, and all-cause diagnoses coded based on the International Classification of Diseases, 9th Revision, Clinical Modification (ICD-9-CM), since 1993 across outpatient, inpatient, and emergency settings for research and auditing purposes in the public sector. The quality and accuracy of CDARS have been demonstrated in population-based studies on COVID-19 [21, 22] and depression [23, 24]. In Hong Kong, the public healthcare is heavily subsidized at a highly affordable price, while the private sector is financed mainly by non-compulsory medical insurance and out-of-pocket payments. The Hospital Authority thus manages 76% of chronic medical conditions including mental health illnesses despite a dual-track public and private system [25].

### Study design and participants

This study consisted of both a quasi-experimental design with ITS analyses and a population-based retrospective study. We first identified all patients who received new clinical diagnoses of major depressive disorder or dysthymia (ICD-9-CM codes: 296.2, 300.4, 311) between January 2014 and December 2022. Patients aged below 10 were excluded to avoid confusion with maternal depression in the coding system. We performed an ITS analysis to evaluate changes in medically attended depression incidence during 36 quarters of data observations. The data cut point was the first quarter of 2020, leaving 24 quarters as pre-cut points and 12 quarters as post-cut points. ITS analysis is a valuable tool to assess the impact of population-level interventions or major macro-environmental changes and widely used in various health policy assessments [26]. Since patients who received incident diagnoses in different years could have different disease durations and care needs, we divided all patients into nine “incident cohorts” (2014 to 2022 cohorts) based on year of depression incidence. All patients were followed up until the end of 2022 for their service use patterns across outpatient, inpatient, and emergency settings.

An exploratory trend analysis showed that use of healthcare resources was the greatest at the beginning of the disease course before stabilizing. Recognizing this feature, we separately investigated the pandemic impact on the (1) initial and (2) ongoing healthcare service use. Respectively, we compared the rates of healthcare service use during the first calendar year following diagnosis, which potentially represents the most care-demanding phase, among patients newly diagnosed during the pandemic (2020 to 2022, the exposure groups) with those diagnosed before the pandemic (2014 to 2019, the reference groups) using a generalized linear model. To study the ongoing resource utilization among the relatively stable prevalent patients, defined as having a disease duration for at least 3 years by the start of the pandemic (i.e., represented by all patients in the 2014–2016 cohorts), we conducted another ITS analysis to compare their rates of service use before and during the pandemic until the end of 2022. The data points before the third calendar year of diagnosis were excluded in the analysis. The linkage between the three parts of analyses is illustrated in Additional file 1: Figure S1.

### Exposure and outcomes of interest

Our study defined the exposure as the macro-environment with the implementation of containment measures in response to the pandemic. Based on the COVID-19 Stringency Index by the Oxford COVID-19 Government Response Tracker, the Hong Kong government introduced relevant policies since January 2020 and announced the lifting of most mandates by December 2022 [27]. With quarterly data, we operationally defined the exposure period starting from the first quarter of 2020 until December 2022 (the intervention period). The reference period (the pre-pandemic period) was between the first quarter of 2014 and the last quarter of 2019.

The first outcome of interest was quarterly incidence of medically attended depression, defined as the number of patients who received depression diagnosis in the current quarter but without history of depression divided by the local eligible population, with age standardization using 5-year age bands based on the 2021 mid-year population. The second outcome was quarterly or yearly rates of attendance episodes or bed-days by incident cohort and service setting, defined by the total visit episodes or bed-days in the current period divided by the number of patients with depression whose observation period (from their first diagnosis to death or end of study) fell within the same period. We further stratified the outpatient attendance into “all-cause” (all outpatient services) and “psychiatric-related” (psychiatric specialist clinic, day hospital, and community nursing) use. Stratified data were unavailable in the inpatient and emergency settings.

### Statistical analysis

In the ITS analyses, we applied segmented quasi-Poisson regression models since the data showed signs of overdispersion [28]. We included a continuous time variable in quarters, a binary indicator for the pandemic period (the exposure period) to represent level change (immediate effect) and the interaction of the two to measure slope change (gradual effect) [29], offsetting the logarithm of the local population or patients with depression. We adjusted the quarters of the data points to account for seasonality. Residual plots, autocorrelation function, and partial autocorrelation function suggested very little evidence of autocorrelation [28, 30]. We then used Newey-West method to obtain robust standard errors and address autocorrelation up to the largest lag [31, 32]. In the comparison of the initial healthcare service use between patients newly diagnosed during and before the pandemic, we fitted the rates of service use in the year of diagnosis between cohorts using a generalized linear model with negative binomial log link function. The model adjusted for a binary indicator of whether the diagnosis year occurred before or during the pandemic (the exposure period) and offset the logarithm of incident patients with depression in each cohort. In all analyses, we excluded data points related to major local social movements in 2014 and 2019 to address confounding due to changes in socio-political environment [33–35].

### Subgroup and sensitivity analyses

In the ITS analysis to evaluate changes in depression incidence, we further stratified the analysis into three age groups: adolescents (10–24), adults (25–64), and the older population (65 +) to explore whether these population subgroups were differentially susceptible to a new depression diagnosis as a result of the pandemic.

During the first quarter of 2022, there was an unprecedented abrupt increase of SARS-CoV-2 cases due to the Omicron variant, marking the start of “fifth-wave outbreak” in Hong Kong [20]. In contrast to the earlier waves of smaller-scale outbreaks (below 13,000 cumulative cases before 2022), the public healthcare services were overwhelmed at the beginning of this wave, which possibly strained diagnostic capacity and caused the number of depression diagnoses to be lower than usual. We therefore performed sensitivity analyses for the ITS analyses for depression incidence and healthcare service use by adjusting a variable indicating the relevant quarter to validate the results. In addition, since outpatient service reception may be subject to long waiting time, we conducted an additional sensitivity analysis with a 6-month lag for the pandemic period by adding a binary indicator for the transition period and re-defining the pandemic to start from the third quarter of 2020. Lastly, we also performed sensitivity analyses for the pandemic impact on ongoing healthcare resource utilization by changing the defined disease duration of 3 years as stable patients into 2 years.

All data were analyzed using R version 4.0.3 and cross-validated by two investigators.

## Results

Over the 9-year study period, we identified 85,111 patients with new depression diagnosis, who generated 4,433,558 attendance or admission episodes across all diagnosis settings and 1,327,424 inpatient bed-days. For these patients, the mean age was 48.6 (SD:19.8) with 71.6% being female. Detailed demographic characteristics of the patients diagnosed in each year are summarized in Additional file 2: Table S1.

### Incidence of medically attended depression

**Fig. 1:**
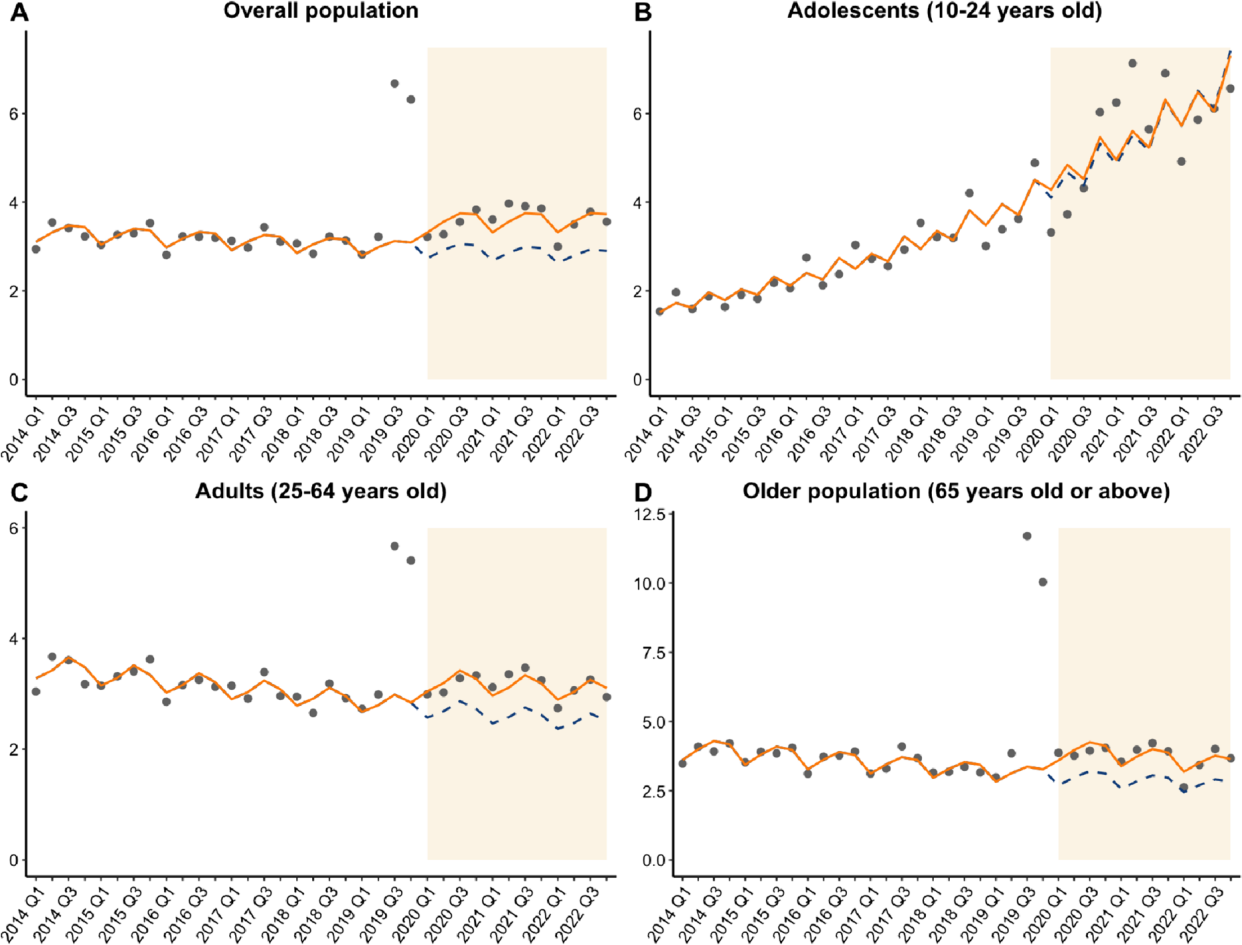
Interrupted time series analysis plot of pandemic impact on depression incidence

Figure 1 illustrates the trends of the observed and model-implied quarterly incidence of medically attended depression between 2014 and 2022. The average quarterly incidence rates were 3.44 and 3.59 per 10,000 population before and during the pandemic (Additional file 2: Table S2), respectively. After adjusting for major social movements, ITS analysis showed a small but marginally significant decline in the population incidence in the pre-pandemic period (risk ratio, RR = 0.995, 95% CI: 0.99–1.00, *p* = 0.042). Since the pandemic, however, there was a significant immediate increase in incidence indicated by level change (RR = 1.21, 95% CI: 1.10–1.33, *p* < 0.001), with a non-significant slope change (Fig. 1A).

Stratifying by age groups, ITS analysis showed a slow but significant decline in incidence in the pre-pandemic period among adults and the older population (RR = 0.99, 95% CI: 0.99–0.99) but a significant increase over the time among adolescents (RR = 1.04, 95% CI: 1.04–1.05) before the pandemic. Since the pandemic, we found significant level increases indicating immediate effects of the pandemic among adults (RR = 1.19, 95% CI: 1.09–1.29) and the older population (RR = 1.33, 95% CI: 1.29–1.38, all *p* < 0.001), but not adolescents. The slope changes remained non-significant in all subgroups (Fig. 1B–D).

In the sensitivity analysis which accounted for the fifth-wave outbreak, we found a similar level change (RR = 1.20, 95% CI: 1.10–1.32, *p* < 0.001) as the main analysis, with a significant but slowly declining pre-pandemic trend (RR = 0.995, 95% CI: 0.990–0.999, *p* = 0.039). Using a 6-month transition window showed a consistent level change (RR = 1.28, 95% CI: 1.22–1.34, *p* < 0.001) and pre-pandemic trend (RR = 0.995, 95% CI: 0.994–0.996, *p* < 0.001). The slope changes in both sensitivity analyses remained non-significant.

### Healthcare service use

**Fig. 2:**
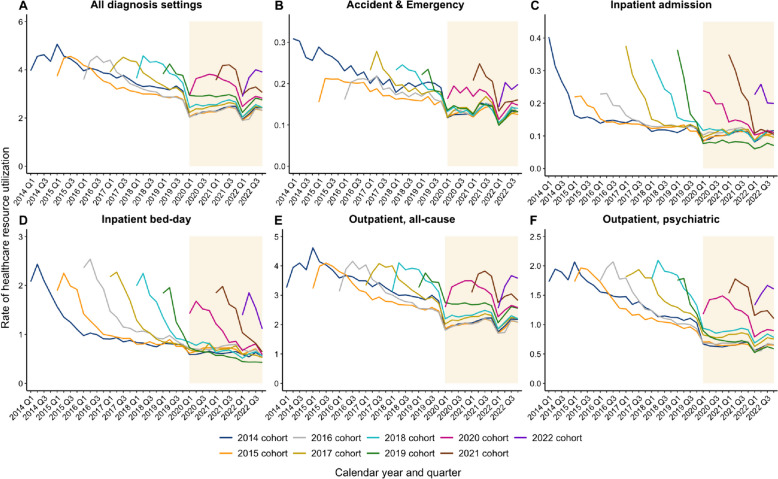
Trend of healthcare resource utilization from 2014 to 2022

In each incident cohort, the patterns followed the natural disease history such that the greatest service demand consistently occurred within the first 2 years of a depression diagnosis, followed by gradual decline subsequently (Fig. 2). During the pandemic, service utilization appeared to decrease further across all diagnosis settings except for inpatient bed-days. All counts and rates of healthcare service use are listed in Additional file 2: Tables S3–S12.

#### Pandemic impact on initial healthcare service use

Table 1 details the rates of healthcare service use in the year of diagnosis stratified by incident cohort and the regression results across diagnosis settings. Annual rates of overall all-cause visits per patient in the year of diagnosis were 10.5 to 10.8 episodes among patients diagnosed between 2015 and 2018, in contrast to 9.0 to 10.2 episodes among those diagnosed between 2020 and 2022. Adjusting for major social movements, the negative binomial model showed that the pandemic was associated with significantly reduced utilization in inpatient bed-days (RR = 0.78, 95% CI: 0.70–0.85), outpatient all-cause visits (RR = 0.89, 95% CI: 0.85–0.93), outpatient psychiatric visits (RR = 0.82, 95% CI: 0.76–0.88), and overall all-cause visits (RR = 0.89, 95% CI: 0.85–0.94, all *p* < 0.001). Being diagnosed during the pandemic was not significantly associated with changes in rates of emergency and inpatient admission episodes.

**Table 1:** Generalized linear regression of pandemic impact on the first-year healthcare resource utilization among cohorts of incident patients

	**A&E**	**Inpatient admission**	**Inpatient bed-day**	**Outpatient, all-cause**	**Outpatient, psychiatric**	**All visits, all-cause**
**First-year rates of healthcare resource utilization**						
2014 incident cohort^a^	0.67	0.67	5.04	9.55	4.5	10.89
2015 incident cohort	0.50	0.48	4.84	9.54	4.55	10.52
2016 incident cohort	0.50	0.49	5.23	9.48	4.62	10.48
2017 incident cohort	0.58	0.63	4.80	9.62	4.58	10.83
2018 incident cohort	0.57	0.63	4.56	9.49	4.73	10.69
2019 incident cohort^a^	0.41	0.40	2.75	7.42	2.91	8.23
2020 incident cohort (*diagnosed in the pandemic period*)	0.45	0.50	3.68	8.03	3.45	8.98
2021 incident cohort (*diagnosed in the pandemic period*)	0.54	0.61	4.12	9.01	4.12	10.15
2022 incident cohort (*diagnosed in the pandemic period*)	0.46	0.51	3.40	8.33	3.81	9.30
**Impact of pandemic on the first-year rate of healthcare resource utilization**^**b**^						
RR	0.891	0.971	0.769	0.887	0.821	0.892
95% CI	0.792–1.001	0.823–1.147	0.699–0.846	0.846–0.930	0.764–0.883	0.846–0.940
* p*-value	0.052	0.728	< 0.001^*^	< 0.001^*^	< 0.001^*^	< 0.001^*^

Healthcare resource utilization was studied only in the first calendar year of diagnosis in each cohort

*Abbreviations*: *A&E* Accident & Emergency, *CI* confidence interval, *RR* risk ratio

^*^Statistical significance at 0.05 in generalized linear regression using negative binomial log-link function

^a^Patients were newly diagnosed in the year of major social movement

^b^Analysis excluded data of the 2014 and 2019 cohorts to adjust for the confounding effect of social movement

#### Pandemic impact on ongoing healthcare service use

**Fig. 3:**
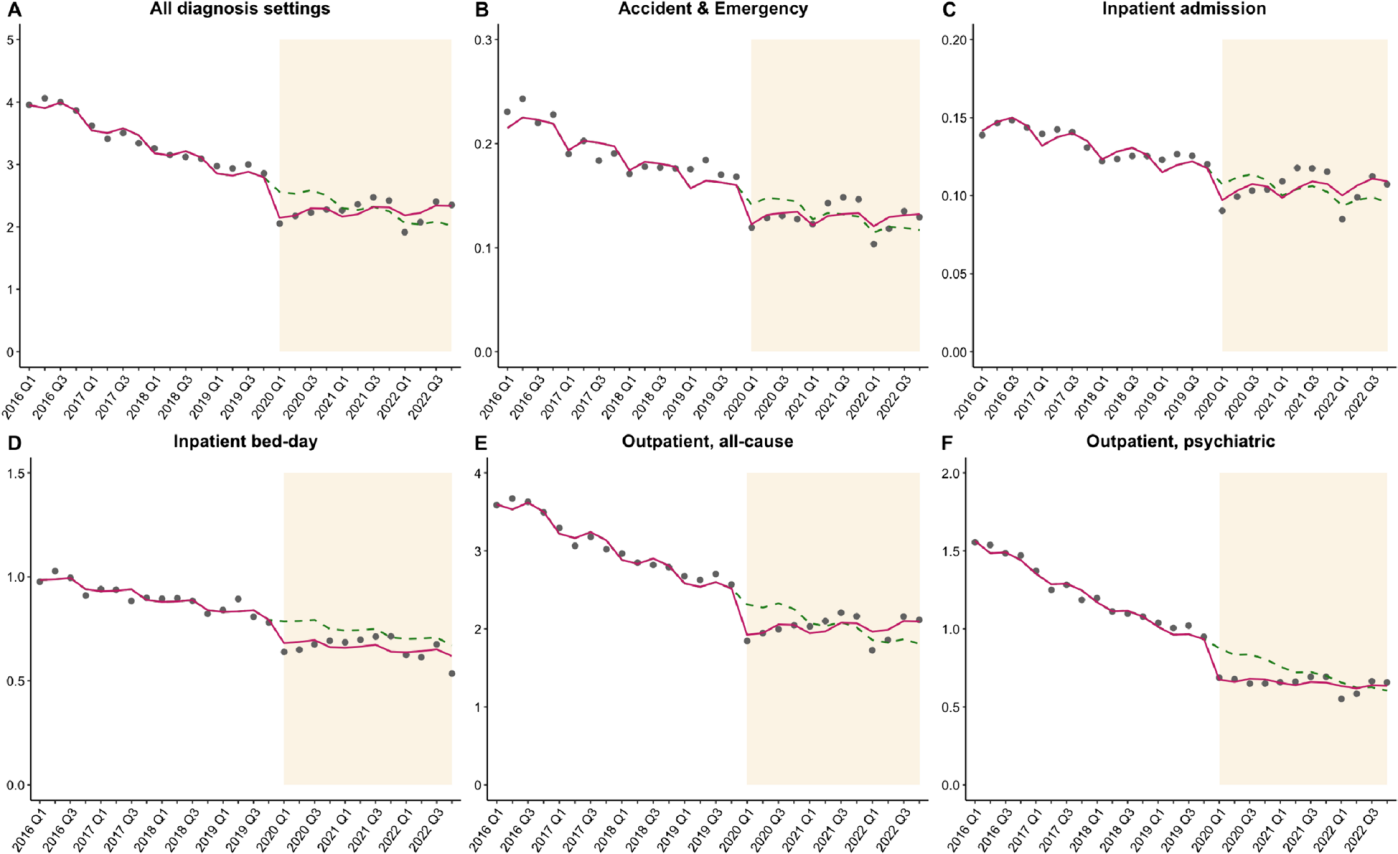
Impact of the pandemic on the ongoing healthcare resource utilization among the 2014–2016 cohorts

For the combined 2014–2016 cohorts, the mean rate of overall all-cause visits counting from their third year of diagnosis was 3.38 episodes per patient in the pre-pandemic period, which dropped to 2.25 episodes per patient in the pandemic period. Adjusting for social movements, the ITS analysis showed significant decreases in the original trends of ongoing service use in all diagnosis settings (RRs ranged from 0.96 to 0.99, all *p* < 0.01) before the pandemic (Table 2 and Fig. 3). When the pandemic began, there were immediate decreases in service use indicated by significant level changes in inpatient admission episodes (RR = 0.91, 95% CI: 0.83–0.99, *p* = 0.024), inpatient bed-days (RR = 0.87, 95% CI: 0.78–0.96, *p* = 0.017), outpatient all-cause visits (RR = 0.83, 95% CI: 0.76–0.91, *p* < 0.001), outpatient psychiatric visits (RR = 0.77, 95% CI: 0.74–0.83, *p* < 0.001), and overall all-cause visits (RR = 0.84, 95% CI: 0.76–0.92, *p* < 0.001), but not emergency visits. Regarding gradual effects, there were significant but small slope changes during the pandemic across all diagnosis settings except inpatient bed-days, with RRs ranging from 1.02 to 1.03, indicating a gradual rebound over time (Table 2 and Fig. 3).

**Table 2:** Interrupted time series results of pandemic impact on the ongoing healthcare resource utilization among the 2014–2016 cohorts

	**RR**	**95% CI**	***p*-value**
**Pre-pandemic trend**			
Accident & Emergency admission	0.974	0.963–0.986	< 0.001^*^
Inpatient admission	0.983	0.979–0.987	< 0.001^*^
Inpatient bed-day	0.986	0.977–0.995	0.007^*^
Outpatient, all-cause	0.973	0.966–0.980	< 0.001^*^
Outpatient, psychiatric	0.964	0.962–0.967	< 0.001^*^
All visits, all-cause	**0.973**	**0.966**–**0.981**	< 0.001^*^
**Level change (immediate effect)**			
Accident & Emergency admission	0.867	0.755–0.996	0.059
Inpatient admission	0.905	0.829–0.987	0.024^*^
Inpatient bed-day	0.867	0.781–0.964	0.017^*^
Outpatient, all-cause	0.831	0.761–0.908	< 0.001^*^
Outpatient, psychiatric	0.770	0.738–0.803	< 0.001^*^
All visits, all-cause	**0.837**	**0.764**–**0.917**	0.001^*^
**Slope change (gradual effect)**			
Accident & Emergency admission	1.024	1.007–1.042	0.006^*^
Inpatient admission	1.021	1.010–1.033	< 0.001^*^
Inpatient bed-day	1.006	0.993–1.019	0.396
Outpatient, all-cause	1.031	1.019–1.042	< 0.001^*^
Outpatient, psychiatric	1.029	1.020–1.037	< 0.001^*^
All visits, all-cause	**1.030**	**1.018**–**1.042**	< 0.001^*^

Healthcare resource utilization was studied starting from the third year of diagnosis. All fitted quasi-Poisson models with seasonality adjustment and excluded the data points relevant to social movements in 2014 and 2019 to adjust for confounding

*Abbreviations*: *CI* confidence interval, *RR* risk ratio

^*^Statistical significance at 0.05 in generalized linear regression using quasi-Poisson model

In the sensitivity analyses accounting for the fifth-wave outbreak and changing definition of disease duration prior to the pandemic, effect sizes were largely consistent with those in the main analysis (Additional file 2: Tables S13–S14).

## Discussion

Using a 9-year population-based study with a quasi-experimental design, we present the immediate and long-term impacts of 3 years of the pandemic on depression burden. We found a 21% immediate increase in incidence of medically attended depression, with 19% and 33% increases among adults and the older population during the 3-year period. There was no significant slope change during the pandemic, indicating a sustained effect towards the end of 2022. Though the pandemic did not affect incidence among adolescents, the incidence had been rising significantly in this subgroup over time even before the pandemic. Despite the increasing overall incidence, patients newly diagnosed during the pandemic used 11% fewer resources in their year of diagnosis than the pre-pandemic patients. Patients with pre-existing depression also had an immediate decrease by 16% in overall all-cause visits, with a positive slope change which suggests a gradual rebound over 3 years.

### Rising incidence of medically attended depression

Our findings are largely consistent with the previous literature that has reported an increased prevalence of depressive mood during the pandemic [7–11]. However, the results from EMR-based studies that focused on clinically confirmed incident diagnoses were mixed. A cohort study based on the UK Biobank reported a 2.0- to 3.1-fold increase in new diagnoses of depressive or anxiety disorders compared to the pre-pandemic period, especially in the first 6 months of the pandemic [36]. Another Israeli time-series analysis observed a 36% increase in depression incidence among youth [37]. Conversely, population-based time-series and cohort studies in the UK found a 28% to 43% decline in recorded depression incidence with a gradual return towards pre-pandemic rates [38, 39]. One explanation for such discrepancies is service disruption during lockdowns that led to under-diagnoses in primary care systems. Alternatively, the nature of social measures may have contributed to the trends differently. Costa-Font et al. highlighted that a “preventive lockdown” when there was low mortality appeared to increase depressive symptoms, but it was the opposite when lockdowns were in a high-mortality context [40]. This echoes with our findings in Hong Kong, where control measures were mostly preventive following the “dynamic zero-COVID” approach while maintaining low case load most of the time.

In our subgroup analysis, we found that adults and the older population were prone to developing depression due to the pandemic, but adolescents were not. However, prior studies tend to report consistent risks across age groups: adults were likely to suffer from job insecurity and increased caregiver responsibilities, older adults were susceptible to prolonged isolation, fear of illness, and grief of losing the loved ones, while adolescents faced school closures, reduced peer interactions, and outdoor activities [37, 41–44]. Between 2014 and 2019, we found the incidence among Hong Kong adolescents was already increasing, with rates doubling within 5 years and overtaking the incidence among adults and the older population. This pre-existing rising trend might explain why the pandemic, despite being an additional risk factor, did not have as comparable impact as in other age groups due to a potential diminishing marginal effect. The earlier rise in adolescent depression may have stemmed from existing contextual forces including social unrest and other unknown stressors [35]. Our findings suggest that resources for depression care among adults and the older population are needed to prepare for future pandemic threats. However, policymakers should be aware of the worrying mental health situation in adolescents. As the rising incidence was minimally linked to the pandemic in this subgroup, it implies that the mental health crisis could persist in the future regardless of the pandemic. Further investigation is needed to confirm the stressors behind the recent trend and ways to reverse the deterioration in adolescent mental health.

### Declining use of healthcare resources

Given the increased demand for depression care during the pandemic, evaluating the pattern in healthcare service use in this critical period is important to identify potential unmet care needs, optimize strategies of service provision, and strengthen the preparedness for future pandemics. Despite the rising incidence, we found that the pandemic substantially reduced the use of inpatient and outpatient services among both newly diagnosed and pre-existing patients. This is consistent with the previous studies in South Africa, South Korea, the United States, and the UK, which estimated 15% to 51% reductions in healthcare resource utilization depending on diagnosis settings [15, 17, 45, 46]. Most of them were conducted during the early phase of the pandemic with a focus on lockdowns. This may explain the generally greater decline in service use compared with our observations for Hong Kong. Among the pre-existing patients, the reductions in service use were unlikely to represent an immediate improvement in depression outcomes but rather the limited capacity of the system even without mobility restriction to access. This also affected the care delivery for the rising number of new patients during the pandemic, who need the greatest care in the first years of diagnosis but accessed less care than historical controls. The findings therefore revealed a suboptimal service provision in response to the extra care demand generated by the pandemic.

In our study, the service types most impacted by the pandemic were the inpatient bed-days for newly diagnosed patients and outpatient psychiatric visits for pre-existing patients. This is consistent with the observation that most inpatient care occurred at the beginning of the disease course, while outpatient follow-ups became more common as patients stabilized. During the pandemic, however, inpatient resources were reserved for outbreak control, leaving the new patients with inadequate service access. Among pre-existing patients, reluctance to visit clinics owing to fear of getting infected may have discouraged them from attending regular appointments [47]. Video consultations for SARS-CoV-2 infected cases have been initiated since July 2022, which led to 214,900 consultations for quarantined patients [48]. “Tele-psychiatry” in the post-pandemic era is worth investigation for its feasibility and effectiveness in extending continuity of care, as it enables follow-ups after hospital discharge and ensures ongoing patient access even without physical attendance.

### Strengths and limitations

One of the major strengths of our study is the use of territory-wide longitudinal data with a large sample size, which allowed a quasi-experimental study design. This enabled us to investigate the population-level impact of the pandemic and validate prior findings from smaller community-based studies. The context of Hong Kong also enabled us to study the longer-term impact of the entire pandemic apart from a focus on lockdowns. When studying healthcare service use, our study differed from previous studies by separating patients into nine incident cohorts before analyzing their rates of service use during the follow-up. This allowed us to differentiate the pandemic impact more clearly on new and pre-existing patients, unlike most of the previous studies.

There are also limitations to our study. Firstly, patients’ decision to seek treatment mediates whether their condition is recorded. Systematic differences between age groups in the propensity to seek treatment during different periods rather than differences in the underlying population-level burden may have driven the trends before and after 2020. Secondly, we were unable to stratify the patterns of service use into all-cause and psychiatric-related use in the emergency and inpatient settings since such information was not available in the raw data. Thirdly, though the public sector provides the majority of local healthcare services, patients may have sought help from private doctors especially when the public healthcare system was overwhelmed at the start of the fifth-wave outbreak, possibly leading to underestimated incidence and healthcare service use. Patients who were diagnosed in private clinics before seeking care in the public sector may also be labeled as incident cases later than actual diagnosis date. We therefore performed sensitivity analyses but found no change in the conclusion. Lastly, the findings represent the mixed overall effect of the pandemic macro-environment, but the current time-series study was unable to disentangle the effects of specific contributing factors.

## Conclusions

Using ITS analyses from a 9-year cohort study, we found a persistent increase in incidence of medically attended depression over the pandemic period in the overall population, adults, and the older population. However, patients newly diagnosed during the pandemic used fewer resources in their first year of diagnosis than pre-pandemic patients. Pre-existing patients also had immediate decreases in healthcare service use following the pandemic in all diagnosis settings, with a gradual rebound over 3 years. Our findings highlight the need to improve the preparedness in mental health resource planning for future public health crises.

### Supplementary Information

**Additional file 1: Figure S1.** Study schema to illustrate the linkage between analyses.

**Additional file 2: Table S1.** Age and sex distribution of patients newly diagnosed with depression between 2014 and 2022. **Table S2.** Quarterly age-standardized incidence and counts of patients newly diagnosed with depression between 2014 to 2022. **Table S3.** Quarterly counts of accident & emergency visit among incident cohorts between 2014 and 2022. **Table S4.** Quarterly counts of inpatient admission among incident cohorts between 2014 and 2022. **Table S5.** Quarterly counts of inpatient stay among incident cohorts between 2014 and 2022. **Table S6.** Quarterly counts of outpatient all-cause visit among incident cohorts between 2014 and 2022. **Table S7.** Quarterly counts of outpatient psychiatric-related visit among incident cohorts between 2014 and 2022. **Table S8.** Quarterly rates of accident & emergency visit among incident cohorts between 2014 and 2022. **Table S9.** Quarterly rates of inpatient admission among incident cohorts between 2014 and 2022. **Table S10.** Quarterly rates of inpatient stay among incident cohorts between 2014 and 2022. **Table S11.** Quarterly rates of outpatient all-cause visit among incident cohorts between 2014 and 2022. **Table S12.** Quarterly rates of outpatient psychiatric-related visit among incident cohorts between 2014 and 2022. **Table S13.** Sensitivity analysis results of ITS analysis of pandemic impact on the ongoing healthcare resource utilization among the 2014-2016 cohorts by adjusting for the fifth-wave outbreak. **Table S14.** Sensitivity analysis results of ITS analysis of pandemic impact on the ongoing healthcare resource utilization among the 2014-2017 cohorts (changing the defined disease duration prior to the pandemic from 3 years to 2 years).

## Data Availability

We are unable to directly share the data used in this study since the data custodian, the Hong Kong Hospital Authority who manages the Clinical Data Analysis and Reporting System (CDARS), has not given permission. However, CDARS data can be accessed via the Hospital Authority Data Sharing Portal for research purpose. The relevant information can be found online (https://www3.ha.org.hk/data).

## Abbreviations

CDARS: Clinical Data Analysis and Reporting System
EMR: Electronic medical records
ITS: Interrupted time-series
RR: Risk ratio
