# Supplementary material for: Impact of COVID-19 pandemic on depression incidence and healthcare service use among patients with depression: an interrupted time-series analysis from a 9-year population-based study
Source: BMC Med. 2024 Apr 22;22:169. doi: 10.1186/s12916-024-03386-z (PMC11034087; doi:10.1186/s12916-024-03386-z)
Supplement: Supplementary file 1 — Additional file 1: Figure S1. Study schema to illustrate the linkage between analyses. [file 12916_2024_3386_MOESM1_ESM.docx]

**Figure S1.** Study schema to illustrate the linkage between analyses

**
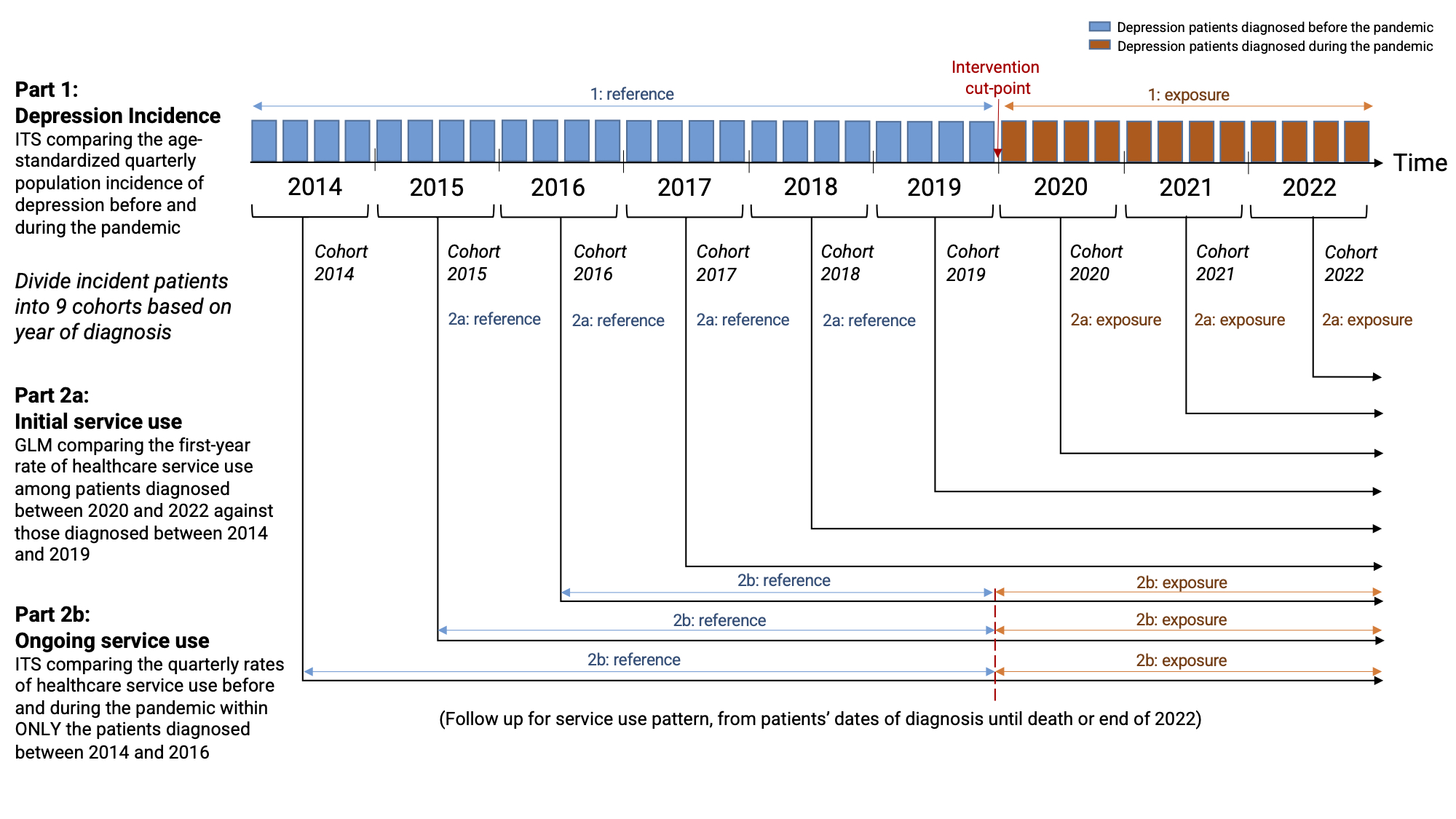
**

Abbreviation: GLM – Generalized linear modelling, ITS – Interrupted time-series.
