# Supplementary material for: Impact of COVID-19 pandemic on depression incidence and healthcare service use among patients with depression: an interrupted time-series analysis from a 9-year population-based study
Source: BMC Med. 2024 Apr 22;22:169. doi: 10.1186/s12916-024-03386-z (PMC11034087; doi:10.1186/s12916-024-03386-z)
Supplement: Supplementary file 2 — Additional file 2: Table S1. Age and sex distribution of patients newly diagnosed with depression between 2014 and 2022. Table S2. Quarterly age-standardized incidence and counts of patients newly diagnosed with depression between 2014 to 2022. Table S3. Quarterly counts of accident & emergency visit among incident cohorts between 2014 and 2022. Table S4. Quarterly counts of inpatient admission among incident cohorts between 2014 and 2022. Table S5. Quarterly counts of inpatient stay among incident cohorts between 2014 and 2022. Table S6. Quarterly counts of outpatient all-cause visit among incident cohorts between 2014 and 2022. Table S7. Quarterly counts of outpatient psychiatric-related visit among incident cohorts between 2014 and 2022. Table S8. Quarterly rates of accident & emergency visit among incident cohorts between 2014 and 2022. Table S9. Quarterly rates of inpatient admission among incident cohorts between 2014 and 2022. Table S10. Quarterly rates of inpatient stay among incident cohorts between 2014 and 2022. Table S11. Quarterly rates of outpatient all-cause visit among incident cohorts between 2014 and 2022. Table S12. Quarterly rates of outpatient psychiatric-related visit among incident cohorts between 2014 and 2022. Table S13. Sensitivity analysis results of ITS analysis of pandemic impact on the ongoing healthcare resource utilization among the 2014-2016 cohorts by adjusting for the fifth-wave outbreak. Table S14. Sensitivity analysis results of ITS analysis of pandemic impact on the ongoing healthcare resource utilization among the 2014-2017 cohorts (changing the defined disease duration prior to the pandemic from 3 years to 2 years). [file 12916_2024_3386_MOESM2_ESM.docx]

**Table S1.** Age and sex distribution of patients newly diagnosed with depression between 2014 and 2022

| **Year** | **N** | **Mean age (SD)** | **Age group** | | | **Sex** | |
| --- | --- | --- | --- | --- | --- | --- | --- |
|  |  |  | **Adolescents**  **(10-24)** | **Adults**  **(25-64)** | **Elderly**  **(65+)** | **Female** | **Male** |
| 2014 | 8608 | 48.8 (18.5) | 826 | 6081 | 1701 | 6144 | 2464 |
| 2015 | 8685 | 49.1 (18.5) | 866 | 6084 | 1735 | 6221 | 2464 |
| 2016 | 8354 | 48.4 (19.2) | 1025 | 5626 | 1703 | 5924 | 2430 |
| 2017 | 8590 | 48.2 (19.5) | 1185 | 5656 | 1749 | 6098 | 2492 |
| 2018 | 8428 | 47.0 (19.9) | 1429 | 5363 | 1636 | 6020 | 2408 |
| 2019 | 12888 | 52.9 (19.3) | 1459 | 7661 | 3768 | 9323 | 3565 |
| 2020 | 9565 | 48.3 (20.2) | 1653 | 5765 | 2147 | 6825 | 2740 |
| 2021 | 10576 | 46.2 (21.1) | 2283 | 6017 | 2276 | 7622 | 2954 |
| 2022 | 9417 | 46.7 (21.0) | 1985 | 5342 | 2090 | 6769 | 2648 |

**Table S2.** Quarterly age-standardized incidence and counts of patients newly diagnosed with depression between 2014 to 2022

| **Quarter** | **Overall** | | **Adolescent (10-24)** | | **Adult (25-65)** | | **Older population (65+)** | |
| --- | --- | --- | --- | --- | --- | --- | --- | --- |
|  | **N** | **Incidence^a^** | **N** | **Incidence^a^** | **N** | **Incidence^a^** | **N** | **Incidence^a^** |
| 2014Q1 | 2027 | 2.94 | 135 | 1.53 | 1387 | 3.04 | 505 | 3.48 |
| 2014Q2 | 2442 | 3.54 | 173 | 1.96 | 1675 | 3.67 | 594 | 4.09 |
| 2014Q3^b^ | 2355 | 3.42 | 140 | 1.59 | 1646 | 3.61 | 569 | 3.92 |
| 2014Q4^b^ | 2225 | 3.23 | 165 | 1.87 | 1449 | 3.18 | 611 | 4.21 |
| 2015Q1 | 2092 | 3.03 | 144 | 1.64 | 1436 | 3.15 | 512 | 3.53 |
| 2015Q2 | 2248 | 3.26 | 168 | 1.91 | 1513 | 3.32 | 567 | 3.91 |
| 2015Q3 | 2273 | 3.30 | 160 | 1.82 | 1553 | 3.40 | 559 | 3.85 |
| 2015Q4 | 2432 | 3.53 | 192 | 2.18 | 1653 | 3.62 | 588 | 4.05 |
| 2016Q1 | 1937 | 2.81 | 181 | 2.06 | 1304 | 2.86 | 452 | 3.11 |
| 2016Q2 | 2225 | 3.23 | 242 | 2.75 | 1441 | 3.16 | 541 | 3.73 |
| 2016Q3 | 2219 | 3.22 | 187 | 2.12 | 1484 | 3.25 | 548 | 3.78 |
| 2016Q4 | 2204 | 3.20 | 209 | 2.37 | 1427 | 3.13 | 568 | 3.91 |
| 2017Q1 | 2156 | 3.13 | 267 | 3.03 | 1436 | 3.15 | 453 | 3.12 |
| 2017Q2 | 2049 | 2.97 | 240 | 2.73 | 1330 | 2.92 | 479 | 3.30 |
| 2017Q3 | 2368 | 3.43 | 225 | 2.56 | 1548 | 3.39 | 595 | 4.10 |
| 2017Q4 | 2144 | 3.11 | 258 | 2.93 | 1351 | 2.96 | 534 | 3.68 |
| 2018Q1 | 2114 | 3.07 | 311 | 3.53 | 1345 | 2.95 | 458 | 3.16 |
| 2018Q2 | 1956 | 2.84 | 283 | 3.21 | 1211 | 2.65 | 462 | 3.18 |
| 2018Q3 | 2222 | 3.22 | 281 | 3.19 | 1453 | 3.18 | 488 | 3.36 |
| 2018Q4 | 2163 | 3.14 | 370 | 4.20 | 1334 | 2.92 | 459 | 3.16 |
| 2019Q1 | 1943 | 2.82 | 265 | 3.01 | 1245 | 2.73 | 433 | 2.98 |
| 2019Q2^b^ | 2219 | 3.22 | 298 | 3.38 | 1363 | 2.99 | 559 | 3.85 |
| 2019Q3^b^ | 4603 | 6.68 | 319 | 3.62 | 2586 | 5.67 | 1698 | 11.7 |
| 2019Q4^b^ | 4354 | 6.32 | 430 | 4.88 | 2467 | 5.41 | 1457 | 10.0 |
| **Mean values (2014-2019)** | **2374** | **3.44** | **235** | **2.67** | **1527** | **3.34** | **612** | **4.22** |
| 2020Q1 | 2219 | 3.22 | 292 | 3.32 | 1365 | 2.99 | 562 | 3.87 |
| 2020Q2 | 2256 | 3.22 | 328 | 3.73 | 1380 | 3.02 | 547 | 3.77 |
| 2020Q3 | 2451 | 3.27 | 380 | 4.32 | 1498 | 3.28 | 573 | 3.95 |
| 2020Q4 | 2640 | 3.55 | 531 | 6.03 | 1520 | 3.33 | 588 | 4.05 |
| 2021Q1 | 2488 | 3.83 | 550 | 6.25 | 1423 | 3.12 | 515 | 3.55 |
| 2021Q2 | 2736 | 3.61 | 628 | 7.13 | 1530 | 3.35 | 578 | 3.98 |
| 2021Q3 | 2694 | 3.97 | 497 | 5.64 | 1584 | 3.47 | 613 | 4.22 |
| 2021Q4 | 2658 | 3.91 | 608 | 6.91 | 1480 | 3.24 | 570 | 3.93 |
| 2022Q1 | 2065 | 3.86 | 433 | 4.92 | 1251 | 2.74 | 381 | 2.62 |
| 2022Q2 | 2411 | 3.00 | 516 | 5.86 | 1397 | 3.06 | 498 | 3.43 |
| 2022Q3 | 2606 | 3.5 | 538 | 6.11 | 1486 | 3.26 | 582 | 4.01 |
| 2022Q4 | 2453 | 3.78 | 578 | 6.56 | 1342 | 2.94 | 533 | 3.67 |
| **Mean values (2020-2022)** | **2473** | **3.59** | **490** | **5.56** | **1438** | **3.15** | **545** | **3.75** |

a. Incidence rate per 10,000 population.

b. Periods relevant to major social movements.

**Table S3.** Quarterly counts of accident & emergency visit among incident cohorts between 2014 and 2022

| **Quarter** | **Year of incident cohort** | | | | | | | | |
| --- | --- | --- | --- | --- | --- | --- | --- | --- | --- |
|  | **2014** | **2015** | **2016** | **2017** | **2018** | **2019** | **2020** | **2021** | **2022** |
| 2014Q1 | 594 | 0 | 0 | 0 | 0 | 0 | 0 | 0 | 0 |
| 2014Q2 | 1285 | 0 | 0 | 0 | 0 | 0 | 0 | 0 | 0 |
| 2014Q3* | 1698 | 0 | 0 | 0 | 0 | 0 | 0 | 0 | 0 |
| 2014Q4* | 2170 | 0 | 0 | 0 | 0 | 0 | 0 | 0 | 0 |
| 2015Q1 | 2429 | 312 | 0 | 0 | 0 | 0 | 0 | 0 | 0 |
| 2015Q2 | 2282 | 880 | 0 | 0 | 0 | 0 | 0 | 0 | 0 |
| 2015Q3 | 2210 | 1323 | 0 | 0 | 0 | 0 | 0 | 0 | 0 |
| 2015Q4 | 2100 | 1808 | 0 | 0 | 0 | 0 | 0 | 0 | 0 |
| 2016Q1 | 1900 | 1733 | 305 | 0 | 0 | 0 | 0 | 0 | 0 |
| 2016Q2 | 1996 | 1707 | 821 | 0 | 0 | 0 | 0 | 0 | 0 |
| 2016Q3 | 1798 | 1690 | 1293 | 0 | 0 | 0 | 0 | 0 | 0 |
| 2016Q4 | 1859 | 1696 | 1747 | 0 | 0 | 0 | 0 | 0 | 0 |
| 2017Q1 | 1627 | 1503 | 1686 | 495 | 0 | 0 | 0 | 0 | 0 |
| 2017Q2 | 1764 | 1558 | 1769 | 1151 | 0 | 0 | 0 | 0 | 0 |
| 2017Q3 | 1589 | 1409 | 1502 | 1513 | 0 | 0 | 0 | 0 | 0 |
| 2017Q4 | 1692 | 1409 | 1521 | 1854 | 0 | 0 | 0 | 0 | 0 |
| 2018Q1 | 1432 | 1330 | 1379 | 1645 | 490 | 0 | 0 | 0 | 0 |
| 2018Q2 | 1521 | 1340 | 1424 | 1652 | 990 | 0 | 0 | 0 | 0 |
| 2018Q3 | 1605 | 1318 | 1329 | 1505 | 1450 | 0 | 0 | 0 | 0 |
| 2018Q4 | 1525 | 1294 | 1401 | 1573 | 1913 | 0 | 0 | 0 | 0 |
| 2019Q1 | 1578 | 1280 | 1331 | 1441 | 1683 | 430 | 0 | 0 | 0 |
| 2019Q2* | 1602 | 1359 | 1416 | 1478 | 1534 | 971 | 0 | 0 | 0 |
| 2019Q3* | 1576 | 1192 | 1263 | 1498 | 1520 | 1571 | 0 | 0 | 0 |
| 2019Q4* | 1486 | 1261 | 1215 | 1418 | 1396 | 2305 | 0 | 0 | 0 |
| 2020Q1 | 914 | 954 | 936 | 962 | 1079 | 1729 | 360 | 0 | 0 |
| 2020Q2 | 968 | 1003 | 1040 | 1099 | 1163 | 1864 | 864 | 0 | 0 |
| 2020Q3 | 970 | 1041 | 1036 | 1146 | 1052 | 1768 | 1218 | 0 | 0 |
| 2020Q4 | 971 | 1015 | 980 | 1146 | 1092 | 1759 | 1816 | 0 | 0 |
| 2021Q1 | 968 | 935 | 940 | 1023 | 981 | 1590 | 1593 | 516 | 0 |
| 2021Q2 | 1171 | 1050 | 1078 | 1227 | 1111 | 1894 | 1749 | 1290 | 0 |
| 2021Q3 | 1142 | 1118 | 1157 | 1347 | 1212 | 1848 | 1683 | 1707 | 0 |
| 2021Q4 | 1106 | 1126 | 1129 | 1215 | 1079 | 1805 | 1503 | 2149 | 0 |
| 2022Q1 | 797 | 806 | 765 | 821 | 840 | 1231 | 1056 | 1386 | 292 |
| 2022Q2 | 895 | 879 | 914 | 916 | 947 | 1411 | 1237 | 1600 | 890 |
| 2022Q3 | 997 | 982 | 1078 | 1006 | 1116 | 1679 | 1417 | 1612 | 1287 |
| 2022Q4 | 992 | 959 | 969 | 1034 | 1100 | 1618 | 1358 | 1651 | 1847 |

*Periods relevant to major social movements.

**Table S4.** Quarterly counts of inpatient admission among incident cohorts between 2014 and 2022

| **Quarter** | **Year of incident cohort** | | | | | | | | |
| --- | --- | --- | --- | --- | --- | --- | --- | --- | --- |
|  | **2014** | **2015** | **2016** | **2017** | **2018** | **2019** | **2020** | **2021** | **2022** |
| 2014Q1 | 776 | 0 | 0 | 0 | 0 | 0 | 0 | 0 | 0 |
| 2014Q2 | 1329 | 0 | 0 | 0 | 0 | 0 | 0 | 0 | 0 |
| 2014Q3* | 1724 | 0 | 0 | 0 | 0 | 0 | 0 | 0 | 0 |
| 2014Q4* | 1949 | 0 | 0 | 0 | 0 | 0 | 0 | 0 | 0 |
| 2015Q1 | 1378 | 442 | 0 | 0 | 0 | 0 | 0 | 0 | 0 |
| 2015Q2 | 1289 | 920 | 0 | 0 | 0 | 0 | 0 | 0 | 0 |
| 2015Q3 | 1318 | 1215 | 0 | 0 | 0 | 0 | 0 | 0 | 0 |
| 2015Q4 | 1259 | 1593 | 0 | 0 | 0 | 0 | 0 | 0 | 0 |
| 2016Q1 | 1144 | 1298 | 432 | 0 | 0 | 0 | 0 | 0 | 0 |
| 2016Q2 | 1202 | 1202 | 932 | 0 | 0 | 0 | 0 | 0 | 0 |
| 2016Q3 | 1214 | 1205 | 1190 | 0 | 0 | 0 | 0 | 0 | 0 |
| 2016Q4 | 1171 | 1138 | 1581 | 0 | 0 | 0 | 0 | 0 | 0 |
| 2017Q1 | 1139 | 1158 | 1336 | 801 | 0 | 0 | 0 | 0 | 0 |
| 2017Q2 | 1201 | 1133 | 1225 | 1191 | 0 | 0 | 0 | 0 | 0 |
| 2017Q3 | 1172 | 1126 | 1179 | 1580 | 0 | 0 | 0 | 0 | 0 |
| 2017Q4 | 1058 | 1071 | 1078 | 1818 | 0 | 0 | 0 | 0 | 0 |
| 2018Q1 | 911 | 1009 | 1034 | 1275 | 704 | 0 | 0 | 0 | 0 |
| 2018Q2 | 945 | 1015 | 1017 | 1214 | 1163 | 0 | 0 | 0 | 0 |
| 2018Q3 | 942 | 1030 | 1038 | 1099 | 1516 | 0 | 0 | 0 | 0 |
| 2018Q4 | 914 | 1026 | 1060 | 1090 | 1908 | 0 | 0 | 0 | 0 |
| 2019Q1 | 872 | 1047 | 1016 | 1072 | 1303 | 708 | 0 | 0 | 0 |
| 2019Q2* | 939 | 1078 | 992 | 1045 | 1219 | 1159 | 0 | 0 | 0 |
| 2019Q3* | 1037 | 923 | 1011 | 1111 | 1186 | 1440 | 0 | 0 | 0 |
| 2019Q4* | 952 | 915 | 966 | 1028 | 1173 | 1809 | 0 | 0 | 0 |
| 2020Q1 | 668 | 660 | 794 | 780 | 951 | 980 | 527 | 0 | 0 |
| 2020Q2 | 698 | 763 | 866 | 839 | 994 | 1020 | 1030 | 0 | 0 |
| 2020Q3 | 784 | 786 | 840 | 900 | 954 | 994 | 1362 | 0 | 0 |
| 2020Q4 | 778 | 798 | 845 | 980 | 951 | 1100 | 1885 | 0 | 0 |
| 2021Q1 | 794 | 831 | 907 | 866 | 821 | 988 | 1349 | 869 | 0 |
| 2021Q2 | 859 | 931 | 931 | 914 | 939 | 1034 | 1402 | 1568 | 0 |
| 2021Q3 | 871 | 879 | 956 | 961 | 939 | 1010 | 1353 | 1834 | 0 |
| 2021Q4 | 858 | 904 | 884 | 958 | 852 | 969 | 1252 | 2154 | 0 |
| 2022Q1 | 617 | 671 | 654 | 671 | 660 | 754 | 956 | 1123 | 460 |
| 2022Q2 | 707 | 835 | 706 | 784 | 784 | 814 | 1004 | 1243 | 1135 |
| 2022Q3 | 852 | 805 | 885 | 802 | 895 | 958 | 1076 | 1161 | 1393 |
| 2022Q4 | 871 | 801 | 749 | 750 | 875 | 866 | 1017 | 1085 | 1857 |

*Periods relevant to major social movements.

**Table S5.** Quarterly counts of inpatient stay among incident cohorts between 2014 and 2022

| **Quarter** | **Year of incident cohort** | | | | | | | | |
| --- | --- | --- | --- | --- | --- | --- | --- | --- | --- |
|  | **2014** | **2015** | **2016** | **2017** | **2018** | **2019** | **2020** | **2021** | **2022** |
| 2014Q1 | 3999 | 0 | 0 | 0 | 0 | 0 | 0 | 0 | 0 |
| 2014Q2 | 10322 | 0 | 0 | 0 | 0 | 0 | 0 | 0 | 0 |
| 2014Q3* | 13533 | 0 | 0 | 0 | 0 | 0 | 0 | 0 | 0 |
| 2014Q4* | 15534 | 0 | 0 | 0 | 0 | 0 | 0 | 0 | 0 |
| 2015Q1 | 13192 | 3800 | 0 | 0 | 0 | 0 | 0 | 0 | 0 |
| 2015Q2 | 11307 | 9317 | 0 | 0 | 0 | 0 | 0 | 0 | 0 |
| 2015Q3 | 10386 | 12479 | 0 | 0 | 0 | 0 | 0 | 0 | 0 |
| 2015Q4 | 9153 | 16424 | 0 | 0 | 0 | 0 | 0 | 0 | 0 |
| 2016Q1 | 8044 | 12070 | 4467 | 0 | 0 | 0 | 0 | 0 | 0 |
| 2016Q2 | 8430 | 10780 | 10257 | 0 | 0 | 0 | 0 | 0 | 0 |
| 2016Q3 | 8141 | 9601 | 13079 | 0 | 0 | 0 | 0 | 0 | 0 |
| 2016Q4 | 7423 | 8364 | 15877 | 0 | 0 | 0 | 0 | 0 | 0 |
| 2017Q1 | 7341 | 8152 | 12036 | 4642 | 0 | 0 | 0 | 0 | 0 |
| 2017Q2 | 7546 | 7836 | 10967 | 9406 | 0 | 0 | 0 | 0 | 0 |
| 2017Q3 | 6831 | 7607 | 9272 | 12766 | 0 | 0 | 0 | 0 | 0 |
| 2017Q4 | 7033 | 7588 | 9023 | 14414 | 0 | 0 | 0 | 0 | 0 |
| 2018Q1 | 6688 | 6575 | 8389 | 10900 | 4198 | 0 | 0 | 0 | 0 |
| 2018Q2 | 6583 | 6562 | 8482 | 9323 | 9063 | 0 | 0 | 0 | 0 |
| 2018Q3 | 6194 | 6922 | 8126 | 8603 | 11218 | 0 | 0 | 0 | 0 |
| 2018Q4 | 5903 | 6458 | 7322 | 7513 | 13915 | 0 | 0 | 0 | 0 |
| 2019Q1 | 6449 | 6469 | 7140 | 7005 | 11182 | 3579 | 0 | 0 | 0 |
| 2019Q2* | 6351 | 7225 | 7660 | 6814 | 8995 | 8095 | 0 | 0 | 0 |
| 2019Q3* | 6261 | 6083 | 6796 | 6639 | 7553 | 10742 | 0 | 0 | 0 |
| 2019Q4* | 6242 | 6014 | 6141 | 6288 | 7310 | 13014 | 0 | 0 | 0 |
| 2020Q1 | 4574 | 4889 | 5541 | 5590 | 6864 | 9290 | 3150 | 0 | 0 |
| 2020Q2 | 4576 | 5220 | 5416 | 5266 | 6320 | 8544 | 7458 | 0 | 0 |
| 2020Q3 | 4798 | 5691 | 5268 | 5326 | 6830 | 8153 | 10445 | 0 | 0 |
| 2020Q4 | 5070 | 5740 | 5299 | 6651 | 6555 | 8088 | 14113 | 0 | 0 |
| 2021Q1 | 4726 | 5694 | 5464 | 5416 | 5062 | 7165 | 11484 | 4597 | 0 |
| 2021Q2 | 4663 | 5625 | 5825 | 5592 | 5323 | 7180 | 10056 | 10300 | 0 |
| 2021Q3 | 4766 | 5680 | 5964 | 5628 | 5429 | 6670 | 7809 | 12675 | 0 |
| 2021Q4 | 4949 | 5412 | 6023 | 6122 | 4858 | 6447 | 8036 | 16000 | 0 |
| 2022Q1 | 4468 | 4574 | 5250 | 4747 | 4082 | 5500 | 6259 | 10757 | 2840 |
| 2022Q2 | 4093 | 5012 | 4824 | 4665 | 4782 | 5364 | 6937 | 9568 | 8121 |
| 2022Q3 | 4730 | 5421 | 5135 | 4514 | 5108 | 5372 | 7449 | 8410 | 10649 |
| 2022Q4 | 4083 | 4027 | 3955 | 4133 | 4467 | 5276 | 5394 | 6583 | 10387 |

Presented counts represent the number of bed-days. *Periods relevant to major social movements.

**Table S6.** Quarterly counts of outpatient all-cause visit among incident cohorts between 2014 and 2022

| **Quarter** | **Year of incident cohort** | | | | | | | | |
| --- | --- | --- | --- | --- | --- | --- | --- | --- | --- |
|  | **2014** | **2015** | **2016** | **2017** | **2018** | **2019** | **2020** | **2021** | **2022** |
| 2014Q1 | 6268 | 0 | 0 | 0 | 0 | 0 | 0 | 0 | 0 |
| 2014Q2 | 16723 | 0 | 0 | 0 | 0 | 0 | 0 | 0 | 0 |
| 2014Q3* | 26419 | 0 | 0 | 0 | 0 | 0 | 0 | 0 | 0 |
| 2014Q4* | 32784 | 0 | 0 | 0 | 0 | 0 | 0 | 0 | 0 |
| 2015Q1 | 38827 | 6498 | 0 | 0 | 0 | 0 | 0 | 0 | 0 |
| 2015Q2 | 34646 | 16507 | 0 | 0 | 0 | 0 | 0 | 0 | 0 |
| 2015Q3 | 33533 | 25729 | 0 | 0 | 0 | 0 | 0 | 0 | 0 |
| 2015Q4 | 31920 | 34135 | 0 | 0 | 0 | 0 | 0 | 0 | 0 |
| 2016Q1 | 29561 | 32330 | 5939 | 0 | 0 | 0 | 0 | 0 | 0 |
| 2016Q2 | 30135 | 31212 | 15730 | 0 | 0 | 0 | 0 | 0 | 0 |
| 2016Q3 | 29671 | 28602 | 25541 | 0 | 0 | 0 | 0 | 0 | 0 |
| 2016Q4 | 28478 | 26531 | 32001 | 0 | 0 | 0 | 0 | 0 | 0 |
| 2017Q1 | 28357 | 25844 | 32990 | 7111 | 0 | 0 | 0 | 0 | 0 |
| 2017Q2 | 26607 | 23618 | 28680 | 15637 | 0 | 0 | 0 | 0 | 0 |
| 2017Q3 | 27588 | 24320 | 27252 | 26233 | 0 | 0 | 0 | 0 | 0 |
| 2017Q4 | 26142 | 22986 | 24737 | 33616 | 0 | 0 | 0 | 0 | 0 |
| 2018Q1 | 24925 | 22794 | 24041 | 33727 | 6593 | 0 | 0 | 0 | 0 |
| 2018Q2 | 23904 | 21931 | 22838 | 29643 | 16559 | 0 | 0 | 0 | 0 |
| 2018Q3 | 23939 | 21736 | 22008 | 28050 | 24099 | 0 | 0 | 0 | 0 |
| 2018Q4 | 23492 | 21606 | 21713 | 26312 | 32727 | 0 | 0 | 0 | 0 |
| 2019Q1 | 23078 | 20683 | 20058 | 25005 | 32112 | 6344 | 0 | 0 | 0 |
| 2019Q2* | 22399 | 20120 | 19919 | 23506 | 28879 | 15551 | 0 | 0 | 0 |
| 2019Q3* | 23534 | 20737 | 19749 | 24024 | 27998 | 29739 | 0 | 0 | 0 |
| 2019Q4* | 21950 | 19711 | 18901 | 22337 | 25524 | 43992 | 0 | 0 | 0 |
| 2020Q1 | 14465 | 14838 | 14003 | 16456 | 17923 | 35063 | 5728 | 0 | 0 |
| 2020Q2 | 14979 | 15695 | 14891 | 17493 | 18847 | 34377 | 14580 | 0 | 0 |
| 2020Q3 | 15603 | 15626 | 15334 | 17322 | 18301 | 34258 | 23352 | 0 | 0 |
| 2020Q4 | 15754 | 16015 | 15804 | 17938 | 18651 | 34566 | 33127 | 0 | 0 |
| 2021Q1 | 15733 | 15915 | 15451 | 18269 | 18625 | 33666 | 32956 | 7695 | 0 |
| 2021Q2 | 16340 | 16358 | 15800 | 18257 | 19319 | 33682 | 30935 | 19375 | 0 |
| 2021Q3 | 16881 | 17152 | 16789 | 18908 | 19870 | 34227 | 30001 | 30025 | 0 |
| 2021Q4 | 16910 | 16537 | 16154 | 18388 | 19198 | 32944 | 28193 | 38181 | 0 |
| 2022Q1 | 13303 | 13291 | 12864 | 14653 | 14743 | 25611 | 21111 | 28616 | 5201 |
| 2022Q2 | 14700 | 14510 | 12988 | 16138 | 16486 | 28934 | 23071 | 30626 | 14501 |
| 2022Q3 | 16442 | 16354 | 16044 | 17593 | 18179 | 32238 | 24487 | 31328 | 25401 |
| 2022Q4 | 16269 | 15928 | 15498 | 17233 | 17262 | 31335 | 23750 | 28999 | 33335 |

*Periods relevant to major social movements.

**Table S7.** Quarterly counts of outpatient psychiatric-related visit among incident cohorts between 2014 and 2022

| **Quarter** | **Year of incident cohort** | | | | | | | | |
| --- | --- | --- | --- | --- | --- | --- | --- | --- | --- |
|  | **2014** | **2015** | **2016** | **2017** | **2018** | **2019** | **2020** | **2021** | **2022** |
| 2014Q1 | 3329 | 0 | 0 | 0 | 0 | 0 | 0 | 0 | 0 |
| 2014Q2 | 8252 | 0 | 0 | 0 | 0 | 0 | 0 | 0 | 0 |
| 2014Q3* | 12204 | 0 | 0 | 0 | 0 | 0 | 0 | 0 | 0 |
| 2014Q4* | 14928 | 0 | 0 | 0 | 0 | 0 | 0 | 0 | 0 |
| 2015Q1 | 17403 | 3483 | 0 | 0 | 0 | 0 | 0 | 0 | 0 |
| 2015Q2 | 15391 | 8141 | 0 | 0 | 0 | 0 | 0 | 0 | 0 |
| 2015Q3 | 14424 | 12184 | 0 | 0 | 0 | 0 | 0 | 0 | 0 |
| 2015Q4 | 13820 | 15697 | 0 | 0 | 0 | 0 | 0 | 0 | 0 |
| 2016Q1 | 12817 | 14676 | 3313 | 0 | 0 | 0 | 0 | 0 | 0 |
| 2016Q2 | 12619 | 13274 | 7940 | 0 | 0 | 0 | 0 | 0 | 0 |
| 2016Q3 | 12144 | 11984 | 12730 | 0 | 0 | 0 | 0 | 0 | 0 |
| 2016Q4 | 11985 | 10712 | 14629 | 0 | 0 | 0 | 0 | 0 | 0 |
| 2017Q1 | 12004 | 10561 | 14644 | 3871 | 0 | 0 | 0 | 0 | 0 |
| 2017Q2 | 10869 | 9621 | 12649 | 7735 | 0 | 0 | 0 | 0 | 0 |
| 2017Q3 | 11200 | 9739 | 11371 | 12455 | 0 | 0 | 0 | 0 | 0 |
| 2017Q4 | 10365 | 8931 | 10107 | 15251 | 0 | 0 | 0 | 0 | 0 |
| 2018Q1 | 9915 | 9144 | 9908 | 15131 | 3726 | 0 | 0 | 0 | 0 |
| 2018Q2 | 9046 | 8617 | 9128 | 12661 | 8456 | 0 | 0 | 0 | 0 |
| 2018Q3 | 9121 | 8499 | 8728 | 11650 | 11894 | 0 | 0 | 0 | 0 |
| 2018Q4 | 8904 | 8358 | 8565 | 11029 | 15749 | 0 | 0 | 0 | 0 |
| 2019Q1 | 8863 | 7996 | 7934 | 10702 | 15243 | 3412 | 0 | 0 | 0 |
| 2019Q2* | 8432 | 7532 | 7944 | 9993 | 13333 | 7398 | 0 | 0 | 0 |
| 2019Q3* | 8683 | 7694 | 7789 | 9784 | 12378 | 11544 | 0 | 0 | 0 |
| 2019Q4* | 8057 | 7075 | 7235 | 9034 | 10857 | 15195 | 0 | 0 | 0 |
| 2020Q1 | 5221 | 5596 | 5312 | 6719 | 7638 | 11467 | 2603 | 0 | 0 |
| 2020Q2 | 4947 | 5618 | 5278 | 6533 | 7399 | 10127 | 6335 | 0 | 0 |
| 2020Q3 | 4853 | 5178 | 5114 | 6278 | 6917 | 9516 | 9936 | 0 | 0 |
| 2020Q4 | 4764 | 5080 | 5266 | 6326 | 7094 | 9216 | 14131 | 0 | 0 |
| 2021Q1 | 4931 | 5093 | 5236 | 6704 | 7179 | 8950 | 13330 | 3814 | 0 |
| 2021Q2 | 4942 | 5104 | 5238 | 6695 | 7268 | 8796 | 11905 | 9249 | 0 |
| 2021Q3 | 5243 | 5262 | 5442 | 6861 | 7504 | 9085 | 11529 | 13354 | 0 |
| 2021Q4 | 5345 | 5175 | 5382 | 6652 | 7284 | 8645 | 10687 | 17123 | 0 |
| 2022Q1 | 4225 | 4187 | 4192 | 4986 | 5475 | 6516 | 7365 | 12005 | 2708 |
| 2022Q2 | 4566 | 4556 | 4148 | 5491 | 6034 | 7174 | 8062 | 12631 | 6659 |
| 2022Q3 | 4966 | 5037 | 5041 | 6098 | 6622 | 7691 | 8454 | 12722 | 11544 |
| 2022Q4 | 4916 | 4981 | 4927 | 5907 | 6167 | 7227 | 8273 | 11339 | 14975 |

*Periods relevant to major social movements.

**Table S8.** Quarterly rates of accident & emergency visit among incident cohorts between 2014 and 2022

| **Quarter** | **Year of incident cohort** | | | | | | | | |
| --- | --- | --- | --- | --- | --- | --- | --- | --- | --- |
|  | **2014** | **2015** | **2016** | **2017** | **2018** | **2019** | **2020** | **2021** | **2022** |
| 2014Q1 | 0.31 | 0 | 0 | 0 | 0 | 0 | 0 | 0 | 0 |
| 2014Q2 | 0.30 | 0 | 0 | 0 | 0 | 0 | 0 | 0 | 0 |
| 2014Q3* | 0.26 | 0 | 0 | 0 | 0 | 0 | 0 | 0 | 0 |
| 2014Q4* | 0.26 | 0 | 0 | 0 | 0 | 0 | 0 | 0 | 0 |
| 2015Q1 | 0.29 | 0.16 | 0 | 0 | 0 | 0 | 0 | 0 | 0 |
| 2015Q2 | 0.27 | 0.21 | 0 | 0 | 0 | 0 | 0 | 0 | 0 |
| 2015Q3 | 0.27 | 0.21 | 0 | 0 | 0 | 0 | 0 | 0 | 0 |
| 2015Q4 | 0.25 | 0.21 | 0 | 0 | 0 | 0 | 0 | 0 | 0 |
| 2016Q1 | 0.23 | 0.20 | 0.16 | 0 | 0 | 0 | 0 | 0 | 0 |
| 2016Q2 | 0.24 | 0.20 | 0.2 | 0 | 0 | 0 | 0 | 0 | 0 |
| 2016Q3 | 0.22 | 0.20 | 0.21 | 0 | 0 | 0 | 0 | 0 | 0 |
| 2016Q4 | 0.23 | 0.20 | 0.21 | 0 | 0 | 0 | 0 | 0 | 0 |
| 2017Q1 | 0.20 | 0.18 | 0.21 | 0.23 | 0 | 0 | 0 | 0 | 0 |
| 2017Q2 | 0.22 | 0.19 | 0.22 | 0.28 | 0 | 0 | 0 | 0 | 0 |
| 2017Q3 | 0.20 | 0.17 | 0.19 | 0.24 | 0 | 0 | 0 | 0 | 0 |
| 2017Q4 | 0.21 | 0.17 | 0.19 | 0.22 | 0 | 0 | 0 | 0 | 0 |
| 2018Q1 | 0.18 | 0.16 | 0.17 | 0.19 | 0.23 | 0 | 0 | 0 | 0 |
| 2018Q2 | 0.19 | 0.16 | 0.18 | 0.20 | 0.25 | 0 | 0 | 0 | 0 |
| 2018Q3 | 0.20 | 0.16 | 0.17 | 0.18 | 0.23 | 0 | 0 | 0 | 0 |
| 2018Q4 | 0.19 | 0.16 | 0.18 | 0.19 | 0.23 | 0 | 0 | 0 | 0 |
| 2019Q1 | 0.20 | 0.16 | 0.17 | 0.17 | 0.20 | 0.22 | 0 | 0 | 0 |
| 2019Q2* | 0.20 | 0.17 | 0.18 | 0.18 | 0.19 | 0.23 | 0 | 0 | 0 |
| 2019Q3* | 0.20 | 0.15 | 0.16 | 0.18 | 0.19 | 0.18 | 0 | 0 | 0 |
| 2019Q4* | 0.19 | 0.16 | 0.16 | 0.17 | 0.17 | 0.18 | 0 | 0 | 0 |
| 2020Q1 | 0.12 | 0.12 | 0.12 | 0.12 | 0.13 | 0.14 | 0.16 | 0 | 0 |
| 2020Q2 | 0.12 | 0.13 | 0.13 | 0.14 | 0.14 | 0.15 | 0.19 | 0 | 0 |
| 2020Q3 | 0.13 | 0.13 | 0.13 | 0.14 | 0.13 | 0.14 | 0.18 | 0 | 0 |
| 2020Q4 | 0.13 | 0.13 | 0.13 | 0.14 | 0.14 | 0.14 | 0.19 | 0 | 0 |
| 2021Q1 | 0.13 | 0.12 | 0.12 | 0.13 | 0.12 | 0.13 | 0.17 | 0.21 | 0 |
| 2021Q2 | 0.15 | 0.13 | 0.14 | 0.15 | 0.14 | 0.15 | 0.19 | 0.25 | 0 |
| 2021Q3 | 0.15 | 0.14 | 0.15 | 0.17 | 0.15 | 0.15 | 0.18 | 0.22 | 0 |
| 2021Q4 | 0.15 | 0.14 | 0.15 | 0.15 | 0.14 | 0.14 | 0.16 | 0.21 | 0 |
| 2022Q1 | 0.11 | 0.10 | 0.10 | 0.10 | 0.11 | 0.10 | 0.11 | 0.13 | 0.14 |
| 2022Q2 | 0.12 | 0.11 | 0.12 | 0.12 | 0.12 | 0.11 | 0.13 | 0.15 | 0.20 |
| 2022Q3 | 0.13 | 0.13 | 0.14 | 0.13 | 0.14 | 0.14 | 0.15 | 0.16 | 0.19 |
| 2022Q4 | 0.13 | 0.13 | 0.13 | 0.13 | 0.14 | 0.13 | 0.15 | 0.16 | 0.2 |

Rate of accident & emergency visit refers to the cumulative number of attendance episode in the accident & emergency setting in the current quarter per observable patient in the same quarter. *Periods relevant to major social movements.

**Table S9.** Quarterly rates of inpatient admission among incident cohorts between 2014 and 2022

| **Quarter** | **Year of incident cohort** | | | | | | | | |
| --- | --- | --- | --- | --- | --- | --- | --- | --- | --- |
|  | **2014** | **2015** | **2016** | **2017** | **2018** | **2019** | **2020** | **2021** | **2022** |
| 2014Q1 | 0.40 | 0 | 0 | 0 | 0 | 0 | 0 | 0 | 0 |
| 2014Q2 | 0.31 | 0 | 0 | 0 | 0 | 0 | 0 | 0 | 0 |
| 2014Q3* | 0.27 | 0 | 0 | 0 | 0 | 0 | 0 | 0 | 0 |
| 2014Q4* | 0.23 | 0 | 0 | 0 | 0 | 0 | 0 | 0 | 0 |
| 2015Q1 | 0.16 | 0.22 | 0 | 0 | 0 | 0 | 0 | 0 | 0 |
| 2015Q2 | 0.15 | 0.22 | 0 | 0 | 0 | 0 | 0 | 0 | 0 |
| 2015Q3 | 0.16 | 0.19 | 0 | 0 | 0 | 0 | 0 | 0 | 0 |
| 2015Q4 | 0.15 | 0.19 | 0 | 0 | 0 | 0 | 0 | 0 | 0 |
| 2016Q1 | 0.14 | 0.15 | 0.23 | 0 | 0 | 0 | 0 | 0 | 0 |
| 2016Q2 | 0.15 | 0.14 | 0.23 | 0 | 0 | 0 | 0 | 0 | 0 |
| 2016Q3 | 0.15 | 0.14 | 0.19 | 0 | 0 | 0 | 0 | 0 | 0 |
| 2016Q4 | 0.14 | 0.14 | 0.19 | 0 | 0 | 0 | 0 | 0 | 0 |
| 2017Q1 | 0.14 | 0.14 | 0.16 | 0.38 | 0 | 0 | 0 | 0 | 0 |
| 2017Q2 | 0.15 | 0.14 | 0.15 | 0.29 | 0 | 0 | 0 | 0 | 0 |
| 2017Q3 | 0.15 | 0.14 | 0.15 | 0.25 | 0 | 0 | 0 | 0 | 0 |
| 2017Q4 | 0.13 | 0.13 | 0.13 | 0.21 | 0 | 0 | 0 | 0 | 0 |
| 2018Q1 | 0.11 | 0.12 | 0.13 | 0.15 | 0.33 | 0 | 0 | 0 | 0 |
| 2018Q2 | 0.12 | 0.12 | 0.13 | 0.14 | 0.29 | 0 | 0 | 0 | 0 |
| 2018Q3 | 0.12 | 0.13 | 0.13 | 0.13 | 0.24 | 0 | 0 | 0 | 0 |
| 2018Q4 | 0.12 | 0.13 | 0.13 | 0.13 | 0.23 | 0 | 0 | 0 | 0 |
| 2019Q1 | 0.11 | 0.13 | 0.13 | 0.13 | 0.16 | 0.36 | 0 | 0 | 0 |
| 2019Q2* | 0.12 | 0.13 | 0.13 | 0.13 | 0.15 | 0.28 | 0 | 0 | 0 |
| 2019Q3* | 0.13 | 0.12 | 0.13 | 0.14 | 0.14 | 0.17 | 0 | 0 | 0 |
| 2019Q4* | 0.12 | 0.11 | 0.12 | 0.13 | 0.14 | 0.14 | 0 | 0 | 0 |
| 2020Q1 | 0.09 | 0.08 | 0.10 | 0.10 | 0.12 | 0.08 | 0.24 | 0 | 0 |
| 2020Q2 | 0.09 | 0.10 | 0.11 | 0.10 | 0.12 | 0.08 | 0.23 | 0 | 0 |
| 2020Q3 | 0.10 | 0.10 | 0.11 | 0.11 | 0.12 | 0.08 | 0.20 | 0 | 0 |
| 2020Q4 | 0.10 | 0.1 | 0.11 | 0.12 | 0.12 | 0.09 | 0.20 | 0 | 0 |
| 2021Q1 | 0.10 | 0.11 | 0.12 | 0.11 | 0.10 | 0.08 | 0.14 | 0.35 | 0 |
| 2021Q2 | 0.11 | 0.12 | 0.12 | 0.11 | 0.12 | 0.08 | 0.15 | 0.30 | 0 |
| 2021Q3 | 0.11 | 0.11 | 0.13 | 0.12 | 0.12 | 0.08 | 0.14 | 0.23 | 0 |
| 2021Q4 | 0.11 | 0.12 | 0.12 | 0.12 | 0.11 | 0.08 | 0.13 | 0.21 | 0 |
| 2022Q1 | 0.08 | 0.09 | 0.09 | 0.08 | 0.08 | 0.06 | 0.10 | 0.11 | 0.23 |
| 2022Q2 | 0.09 | 0.11 | 0.09 | 0.10 | 0.10 | 0.07 | 0.11 | 0.12 | 0.26 |
| 2022Q3 | 0.11 | 0.10 | 0.12 | 0.10 | 0.11 | 0.08 | 0.12 | 0.11 | 0.20 |
| 2022Q4 | 0.12 | 0.10 | 0.10 | 0.10 | 0.11 | 0.07 | 0.11 | 0.11 | 0.20 |

Rate of inpatient admission refers to the cumulative number of hospitalization in the current quarter per observable patient in the same quarter. *Periods relevant to major social movements.

**Table S10.** Quarterly rates of inpatient stay among incident cohorts between 2014 and 2022

| **Quarter** | **Year of incident cohort** | | | | | | | | |
| --- | --- | --- | --- | --- | --- | --- | --- | --- | --- |
|  | **2014** | **2015** | **2016** | **2017** | **2018** | **2019** | **2020** | **2021** | **2022** |
| 2014Q1 | 2.08 | 0 | 0 | 0 | 0 | 0 | 0 | 0 | 0 |
| 2014Q2 | 2.43 | 0 | 0 | 0 | 0 | 0 | 0 | 0 | 0 |
| 2014Q3* | 2.10 | 0 | 0 | 0 | 0 | 0 | 0 | 0 | 0 |
| 2014Q4* | 1.83 | 0 | 0 | 0 | 0 | 0 | 0 | 0 | 0 |
| 2015Q1 | 1.57 | 1.89 | 0 | 0 | 0 | 0 | 0 | 0 | 0 |
| 2015Q2 | 1.35 | 2.25 | 0 | 0 | 0 | 0 | 0 | 0 | 0 |
| 2015Q3 | 1.25 | 1.99 | 0 | 0 | 0 | 0 | 0 | 0 | 0 |
| 2015Q4 | 1.10 | 1.92 | 0 | 0 | 0 | 0 | 0 | 0 | 0 |
| 2016Q1 | 0.98 | 1.42 | 2.36 | 0 | 0 | 0 | 0 | 0 | 0 |
| 2016Q2 | 1.03 | 1.28 | 2.54 | 0 | 0 | 0 | 0 | 0 | 0 |
| 2016Q3 | 1.00 | 1.14 | 2.13 | 0 | 0 | 0 | 0 | 0 | 0 |
| 2016Q4 | 0.91 | 1.00 | 1.93 | 0 | 0 | 0 | 0 | 0 | 0 |
| 2017Q1 | 0.90 | 0.98 | 1.47 | 2.18 | 0 | 0 | 0 | 0 | 0 |
| 2017Q2 | 0.93 | 0.94 | 1.35 | 2.27 | 0 | 0 | 0 | 0 | 0 |
| 2017Q3 | 0.85 | 0.92 | 1.15 | 1.99 | 0 | 0 | 0 | 0 | 0 |
| 2017Q4 | 0.88 | 0.92 | 1.12 | 1.70 | 0 | 0 | 0 | 0 | 0 |
| 2018Q1 | 0.84 | 0.80 | 1.05 | 1.29 | 2.00 | 0 | 0 | 0 | 0 |
| 2018Q2 | 0.83 | 0.80 | 1.07 | 1.11 | 2.25 | 0 | 0 | 0 | 0 |
| 2018Q3 | 0.78 | 0.85 | 1.03 | 1.03 | 1.80 | 0 | 0 | 0 | 0 |
| 2018Q4 | 0.75 | 0.79 | 0.93 | 0.90 | 1.67 | 0 | 0 | 0 | 0 |
| 2019Q1 | 0.82 | 0.80 | 0.91 | 0.85 | 1.35 | 1.84 | 0 | 0 | 0 |
| 2019Q2* | 0.81 | 0.90 | 0.98 | 0.83 | 1.09 | 1.96 | 0 | 0 | 0 |
| 2019Q3* | 0.80 | 0.76 | 0.87 | 0.81 | 0.92 | 1.25 | 0 | 0 | 0 |
| 2019Q4* | 0.80 | 0.75 | 0.79 | 0.77 | 0.89 | 1.02 | 0 | 0 | 0 |
| 2020Q1 | 0.59 | 0.61 | 0.72 | 0.69 | 0.84 | 0.73 | 1.43 | 0 | 0 |
| 2020Q2 | 0.59 | 0.66 | 0.70 | 0.65 | 0.78 | 0.67 | 1.68 | 0 | 0 |
| 2020Q3 | 0.62 | 0.72 | 0.68 | 0.66 | 0.84 | 0.64 | 1.52 | 0 | 0 |
| 2020Q4 | 0.66 | 0.73 | 0.69 | 0.82 | 0.81 | 0.64 | 1.49 | 0 | 0 |
| 2021Q1 | 0.62 | 0.72 | 0.71 | 0.67 | 0.63 | 0.57 | 1.22 | 1.85 | 0 |
| 2021Q2 | 0.61 | 0.72 | 0.76 | 0.70 | 0.66 | 0.57 | 1.07 | 1.98 | 0 |
| 2021Q3 | 0.63 | 0.73 | 0.79 | 0.71 | 0.68 | 0.53 | 0.83 | 1.61 | 0 |
| 2021Q4 | 0.65 | 0.69 | 0.80 | 0.77 | 0.61 | 0.52 | 0.86 | 1.53 | 0 |
| 2022Q1 | 0.59 | 0.59 | 0.70 | 0.60 | 0.51 | 0.44 | 0.67 | 1.03 | 1.40 |
| 2022Q2 | 0.54 | 0.65 | 0.64 | 0.59 | 0.60 | 0.43 | 0.75 | 0.93 | 1.85 |
| 2022Q3 | 0.63 | 0.71 | 0.69 | 0.58 | 0.65 | 0.44 | 0.81 | 0.82 | 1.54 |
| 2022Q4 | 0.55 | 0.53 | 0.53 | 0.53 | 0.57 | 0.43 | 0.59 | 0.64 | 1.11 |

Rate of inpatient stay refers to the cumulative number of bed-days in the current quarter per observable patient in the same quarter. *Periods relevant to major social movements.

**Table S11.** Quarterly rates of outpatient all-cause visit among incident cohorts between 2014 and 2022

| **Quarter** | **Year of incident cohort** | | | | | | | | |
| --- | --- | --- | --- | --- | --- | --- | --- | --- | --- |
|  | **2014** | **2015** | **2016** | **2017** | **2018** | **2019** | **2020** | **2021** | **2022** |
| 2014Q1 | 3.25 | 0 | 0 | 0 | 0 | 0 | 0 | 0 | 0 |
| 2014Q2 | 3.94 | 0 | 0 | 0 | 0 | 0 | 0 | 0 | 0 |
| 2014Q3* | 4.10 | 0 | 0 | 0 | 0 | 0 | 0 | 0 | 0 |
| 2014Q4* | 3.87 | 0 | 0 | 0 | 0 | 0 | 0 | 0 | 0 |
| 2015Q1 | 4.61 | 3.23 | 0 | 0 | 0 | 0 | 0 | 0 | 0 |
| 2015Q2 | 4.14 | 3.99 | 0 | 0 | 0 | 0 | 0 | 0 | 0 |
| 2015Q3 | 4.03 | 4.10 | 0 | 0 | 0 | 0 | 0 | 0 | 0 |
| 2015Q4 | 3.85 | 3.98 | 0 | 0 | 0 | 0 | 0 | 0 | 0 |
| 2016Q1 | 3.59 | 3.80 | 3.14 | 0 | 0 | 0 | 0 | 0 | 0 |
| 2016Q2 | 3.67 | 3.70 | 3.89 | 0 | 0 | 0 | 0 | 0 | 0 |
| 2016Q3 | 3.63 | 3.40 | 4.15 | 0 | 0 | 0 | 0 | 0 | 0 |
| 2016Q4 | 3.49 | 3.17 | 3.88 | 0 | 0 | 0 | 0 | 0 | 0 |
| 2017Q1 | 3.49 | 3.10 | 4.04 | 3.34 | 0 | 0 | 0 | 0 | 0 |
| 2017Q2 | 3.29 | 2.84 | 3.53 | 3.78 | 0 | 0 | 0 | 0 | 0 |
| 2017Q3 | 3.43 | 2.94 | 3.37 | 4.08 | 0 | 0 | 0 | 0 | 0 |
| 2017Q4 | 3.25 | 2.79 | 3.08 | 3.96 | 0 | 0 | 0 | 0 | 0 |
| 2018Q1 | 3.11 | 2.78 | 3.01 | 4.00 | 3.13 | 0 | 0 | 0 | 0 |
| 2018Q2 | 3.00 | 2.68 | 2.88 | 3.54 | 4.10 | 0 | 0 | 0 | 0 |
| 2018Q3 | 3.01 | 2.67 | 2.78 | 3.36 | 3.87 | 0 | 0 | 0 | 0 |
| 2018Q4 | 2.97 | 2.66 | 2.75 | 3.17 | 3.92 | 0 | 0 | 0 | 0 |
| 2019Q1 | 2.92 | 2.55 | 2.55 | 3.03 | 3.87 | 3.26 | 0 | 0 | 0 |
| 2019Q2* | 2.84 | 2.50 | 2.54 | 2.86 | 3.50 | 3.76 | 0 | 0 | 0 |
| 2019Q3* | 3.00 | 2.58 | 2.53 | 2.93 | 3.41 | 3.46 | 0 | 0 | 0 |
| 2019Q4* | 2.81 | 2.47 | 2.43 | 2.73 | 3.12 | 3.43 | 0 | 0 | 0 |
| 2020Q1 | 1.86 | 1.86 | 1.81 | 2.02 | 2.20 | 2.75 | 2.59 | 0 | 0 |
| 2020Q2 | 1.93 | 1.98 | 1.93 | 2.15 | 2.32 | 2.70 | 3.28 | 0 | 0 |
| 2020Q3 | 2.02 | 1.97 | 1.99 | 2.14 | 2.26 | 2.70 | 3.40 | 0 | 0 |
| 2020Q4 | 2.05 | 2.03 | 2.06 | 2.22 | 2.31 | 2.74 | 3.49 | 0 | 0 |
| 2021Q1 | 2.05 | 2.02 | 2.02 | 2.27 | 2.31 | 2.68 | 3.49 | 3.09 | 0 |
| 2021Q2 | 2.14 | 2.09 | 2.07 | 2.28 | 2.41 | 2.69 | 3.29 | 3.72 | 0 |
| 2021Q3 | 2.22 | 2.20 | 2.21 | 2.37 | 2.48 | 2.74 | 3.20 | 3.82 | 0 |
| 2021Q4 | 2.23 | 2.12 | 2.14 | 2.31 | 2.41 | 2.65 | 3.02 | 3.65 | 0 |
| 2022Q1 | 1.76 | 1.71 | 1.71 | 1.85 | 1.85 | 2.06 | 2.27 | 2.75 | 2.56 |
| 2022Q2 | 1.95 | 1.88 | 1.74 | 2.05 | 2.08 | 2.34 | 2.49 | 2.96 | 3.30 |
| 2022Q3 | 2.19 | 2.13 | 2.15 | 2.24 | 2.31 | 2.62 | 2.65 | 3.04 | 3.67 |
| 2022Q4 | 2.18 | 2.08 | 2.09 | 2.20 | 2.20 | 2.55 | 2.58 | 2.82 | 3.58 |

Rate of outpatient all-cause visit refers to the cumulative number of all-cause attendance episode in the outpatient setting in the current quarter per observable patient in the same quarter. *Periods relevant to major social movements.

**Table S12.** Quarterly rates of outpatient psychiatric-related visit among incident cohorts between 2014 and 2022

| **Quarter** | **Year of incident cohort** | | | | | | | | |
| --- | --- | --- | --- | --- | --- | --- | --- | --- | --- |
|  | **2014** | **2015** | **2016** | **2017** | **2018** | **2019** | **2020** | **2021** | **2022** |
| 2014Q1 | 1.73 | 0 | 0 | 0 | 0 | 0 | 0 | 0 | 0 |
| 2014Q2 | 1.95 | 0 | 0 | 0 | 0 | 0 | 0 | 0 | 0 |
| 2014Q3* | 1.89 | 0 | 0 | 0 | 0 | 0 | 0 | 0 | 0 |
| 2014Q4* | 1.76 | 0 | 0 | 0 | 0 | 0 | 0 | 0 | 0 |
| 2015Q1 | 2.07 | 1.73 | 0 | 0 | 0 | 0 | 0 | 0 | 0 |
| 2015Q2 | 1.84 | 1.97 | 0 | 0 | 0 | 0 | 0 | 0 | 0 |
| 2015Q3 | 1.73 | 1.94 | 0 | 0 | 0 | 0 | 0 | 0 | 0 |
| 2015Q4 | 1.67 | 1.83 | 0 | 0 | 0 | 0 | 0 | 0 | 0 |
| 2016Q1 | 1.56 | 1.73 | 1.75 | 0 | 0 | 0 | 0 | 0 | 0 |
| 2016Q2 | 1.54 | 1.57 | 1.96 | 0 | 0 | 0 | 0 | 0 | 0 |
| 2016Q3 | 1.48 | 1.43 | 2.07 | 0 | 0 | 0 | 0 | 0 | 0 |
| 2016Q4 | 1.47 | 1.28 | 1.78 | 0 | 0 | 0 | 0 | 0 | 0 |
| 2017Q1 | 1.48 | 1.27 | 1.79 | 1.82 | 0 | 0 | 0 | 0 | 0 |
| 2017Q2 | 1.34 | 1.16 | 1.56 | 1.87 | 0 | 0 | 0 | 0 | 0 |
| 2017Q3 | 1.39 | 1.18 | 1.41 | 1.94 | 0 | 0 | 0 | 0 | 0 |
| 2017Q4 | 1.29 | 1.08 | 1.26 | 1.80 | 0 | 0 | 0 | 0 | 0 |
| 2018Q1 | 1.24 | 1.11 | 1.24 | 1.79 | 1.77 | 0 | 0 | 0 | 0 |
| 2018Q2 | 1.13 | 1.05 | 1.15 | 1.51 | 2.10 | 0 | 0 | 0 | 0 |
| 2018Q3 | 1.15 | 1.04 | 1.10 | 1.4 | 1.91 | 0 | 0 | 0 | 0 |
| 2018Q4 | 1.12 | 1.03 | 1.09 | 1.33 | 1.89 | 0 | 0 | 0 | 0 |
| 2019Q1 | 1.12 | 0.99 | 1.01 | 1.29 | 1.84 | 1.76 | 0 | 0 | 0 |
| 2019Q2* | 1.07 | 0.93 | 1.01 | 1.21 | 1.62 | 1.79 | 0 | 0 | 0 |
| 2019Q3* | 1.11 | 0.96 | 1.00 | 1.19 | 1.51 | 1.34 | 0 | 0 | 0 |
| 2019Q4* | 1.03 | 0.89 | 0.93 | 1.11 | 1.33 | 1.19 | 0 | 0 | 0 |
| 2020Q1 | 0.67 | 0.70 | 0.69 | 0.82 | 0.94 | 0.90 | 1.18 | 0 | 0 |
| 2020Q2 | 0.64 | 0.71 | 0.68 | 0.80 | 0.91 | 0.80 | 1.43 | 0 | 0 |
| 2020Q3 | 0.63 | 0.65 | 0.66 | 0.78 | 0.85 | 0.75 | 1.45 | 0 | 0 |
| 2020Q4 | 0.62 | 0.64 | 0.69 | 0.78 | 0.88 | 0.73 | 1.49 | 0 | 0 |
| 2021Q1 | 0.64 | 0.65 | 0.68 | 0.83 | 0.89 | 0.71 | 1.41 | 1.53 | 0 |
| 2021Q2 | 0.65 | 0.65 | 0.69 | 0.84 | 0.91 | 0.70 | 1.27 | 1.78 | 0 |
| 2021Q3 | 0.69 | 0.67 | 0.72 | 0.86 | 0.94 | 0.73 | 1.23 | 1.70 | 0 |
| 2021Q4 | 0.70 | 0.66 | 0.71 | 0.84 | 0.91 | 0.69 | 1.14 | 1.64 | 0 |
| 2022Q1 | 0.56 | 0.54 | 0.56 | 0.63 | 0.69 | 0.52 | 0.79 | 1.15 | 1.33 |
| 2022Q2 | 0.61 | 0.59 | 0.55 | 0.70 | 0.76 | 0.58 | 0.87 | 1.22 | 1.52 |
| 2022Q3 | 0.66 | 0.66 | 0.68 | 0.78 | 0.84 | 0.62 | 0.92 | 1.24 | 1.67 |
| 2022Q4 | 0.66 | 0.65 | 0.66 | 0.76 | 0.78 | 0.59 | 0.90 | 1.10 | 1.61 |

Rate of outpatient psychiatric-related visit refers to the cumulative number of attendance episode in the psychiatric day hospital, psychiatric specialist outpatient clinic and community psychiatric nursing services in the current quarter per observable patient in the same quarter. *Periods relevant to major social movements.

**Table S13.** Sensitivity analysis results of ITS analysis of pandemic impact on the ongoing healthcare resource utilization among the 2014-2016 cohorts by adjusting for the fifth-wave outbreak

|  | **RR** | **95%CI** | **p-value** |
| --- | --- | --- | --- |
| **Pre-pandemic trend** | | | |
| Accident & Emergency | 0.974 | 0.964 - 0.984 | <0.001* |
| Inpatient admission | 0.983 | 0.979 - 0.987 | <0.001* |
| Inpatient bed-day | 0.986 | 0.977 - 0.995 | 0.008* |
| Outpatient, all-cause | 0.973 | 0.967 - 0.978 | <0.001* |
| Outpatient, psychiatric | 0.965 | 0.962 - 0.967 | <0.001* |
| All visits, all-cause | **0.973** | **0.968 - 0.979** | **<0.001*** |
| **Level change (immediate effect)** | | | |
| Accident & Emergency | 0.862 | 0.761 - 0.975 | 0.031* |
| Inpatient admission | 0.899 | 0.820 - 0.986 | 0.024* |
| Inpatient bed-day | 0.867 | 0.778 - 0.966 | 0.019* |
| Outpatient, all-cause | 0.827 | 0.773 - 0.885 | <0.001* |
| Outpatient, psychiatric | 0.766 | 0.739 - 0.793 | <0.001* |
| All visits, all-cause | **0.832** | **0.776 - 0.893** | **<0.001*** |
| **Slope change (gradual effect)** | | | |
| Accident & Emergency | 1.029 | 1.013 - 1.045 | <0.003* |
| Inpatient admission | 1.026 | 1.014 - 1.039 | <0.001* |
| Inpatient bed-day | 1.006 | 0.993 - 1.020 | 0.374 |
| Outpatient, all-cause | 1.034 | 1.026 - 1.043 | <0.001* |
| Outpatient, psychiatric | 1.032 | 1.028 - 1.037 | <0.001* |
| All visits, all-cause | **1.034** | **1.025 - 1.043** | **<0.001*** |

Healthcare resource utilization was studied starting from the third year of diagnosis. As a sensitivity analysis, quasi-Poisson models adjusted for the beginning of the fifth wave outbreak to validate the findings in the main analysis. All fitted quasi-Poisson models with seasonality adjustment and excluded the data points relevant to social movements in 2014 and 2019 to adjust for confounding. *Statistical significance at 0.05 in generalized linear regression using quasi-Poisson model. Abbreviations: CI – Confidence interval, RR – Risk ratio.

**Table S14.** Sensitivity analysis results of ITS analysis of pandemic impact on the ongoing healthcare resource utilization among the 2014-2017 cohorts (changing the defined disease duration prior to the pandemic from 3 years to 2 years)

|  | **RR** | **95%CI** | **p-value** |
| --- | --- | --- | --- |
| **Pre-pandemic trend** | | | |
| Accident & Emergency | 0.975 | 0.962 - 0.989 | 0.002* |
| Inpatient admission | 0.984 | 0.979 - 0.989 | <0.001* |
| Inpatient bed-day | 0.986 | 0.975 - 0.997 | 0.751 |
| Outpatient, all-cause | 0.975 | 0.967 - 0.983 | <0.001* |
| Outpatient, psychiatric | 0.968 | 0.962 - 0.973 | <0.001* |
| All visits, all-cause | **0.975** | **0.967 - 0.984** | **<0.001*** |
| **Level change (immediate effect)** | | | |
| Accident & Emergency | 0.872 | 0.749 - 1.016 | 0.096 |
| Inpatient admission | 0.912 | 0.830 - 1.003 | 0.058 |
| Inpatient bed-day | 0.885 | 0.786 - 0.997 | 0.059 |
| Outpatient, all-cause | 0.833 | 0.757 - 0.918 | 0.002* |
| Outpatient, psychiatric | 0.784 | 0.733 - 0.839 | <0.001* |
| All visits, all-cause | **0.839** | **0.759 - 0.927** | **0.003*** |
| **Slope change (gradual effect)** | | | |
| Accident & Emergency | 1.021 | 1.002 - 1.040 | 0.042* |
| Inpatient admission | 1.016 | 1.003 - 1.029 | 0.045* |
| Inpatient bed-day | 1.002 | 0.988 - 1.017 | 0.751 |
| Outpatient, all-cause | 1.028 | 1.015 - 1.040 | <0.001* |
| Outpatient, psychiatric | 1.025 | 1.014 - 1.035 | <0.001* |
| All visits, all-cause | **1.027** | **1.014 - 1.039** | **<0.001*** |

Healthcare resource utilization was studied starting from the third year of diagnosis. As a sensitivity analysis, disease duration of at least three years was modified to two years to validate the findings in the main analysis. All fitted quasi-Poisson models with seasonality adjustment and excluded the data points relevant to social movements in 2014 and 2019 to adjust for confounding. *Statistical significance at 0.05 in generalized linear regression using quasi-Poisson model. Abbreviations: CI – Confidence interval, RR – Risk ratio.
